# Electron microscopy‐based three‐dimensional subcellular imaging of plant male gametophyte

**DOI:** 10.1111/jipb.70143

**Published:** 2026-01-21

**Authors:** Zhiqi Liu, Zizhen Liang, Mengfei Liao, Yixin Huang, Rui Ma, Jiayang Gao, Weiqi Wang, Tao Ni, Philipp S. Erdmann, Liwen Jiang

**Affiliations:** ^1^ Centre for Cell & Developmental Biology and State Key Laboratory of Agrobiotechnology, School of Life Sciences The Chinese University of Hong Kong Hong Kong 999077 China; ^2^ AoE Centre for Organelle Biogenesis and Function, AoE Centre for Plant Vacuole Biology and Biotechnology The Chinese University of Hong Kong Hong Kong 999077 China; ^3^ School of Biomedical Sciences, Li Ka Shing Faculty of Medicine The University of Hong Kong Hong Kong 999077 China; ^4^ Shanghai Nanoport Thermofisher Scientific Shanghai 201203 China; ^5^ Materials Innovation Institute for Life Sciences and Energy (MILES) The University of Hong Kong Shenzhen Institute of Research and Innovation Shenzhen 518048 China; ^6^ Human Technopole Milan 20157 Italy; ^7^ Institute of Plant Molecular Biology and Agricultural Biotechnology The Chinese University of Hong Kong Hong Kong 999077 China; ^8^ CUHK Shenzhen Research Institute Shenzhen 518057 China

**Keywords:** 3D imaging, cryo‐ET, cryo‐lift‐out, dual‐beam FIB‐SEM, volume electron microscopy

## Abstract

Understanding cellular events in three dimensions (3D) is of great importance for the annotation and illustration of biological processes in a contextual way. Imaging techniques based on electron microscopy (EM), such as those derived from scanning electron microscopy (SEM) and transmission electron microscopy (TEM), provide various options to visualize biological samples at scales ranging from cells to macromolecules *in situ*. Recently, a series of cryogenic techniques has brought EM‐based imaging to a new level, enabling specimens to retain their hydrated state throughout the sample preparation and imaging steps, thereby offering a near‐native visualization of cellular events. The application of dual‐beam focused ion beam (FIB)‐SEM to biological samples has enabled high‐resolution reconstructions in 3D and streamlined sample preparation workflows for downstream cryo‐electron tomography (cryo‐ET) imaging. However, applications of these technologies to plant materials are limited due to intrinsic characteristics of plant cells (e.g., non‐adhesive growth, large size with a central vacuole, and the presence of cell walls). For the timely application of dual‐beam FIB‐SEM in three‐dimensional subcellular imaging of plant materials, we have recently tested and developed three major workflows with proof‐of‐concept evidence using developing anthers and *in vitro*‐cultured pollen tubes based on Aquilos 2 Cryo‐FIB, including (1) room‐temperature FIB‐SEM volume imaging, (2) cryo‐lamellae preparation from cell suspension culture or high‐pressure‐frozen organs for cryo‐ET imaging, and (3) cryo‐FIB‐SEM volume imaging, which will facilitate structural studies of plant materials and provide technical guidance for the broader plant cell biology research community.

## Graphical abstract

The Aquilos2 Cryo‐FIB workflows provide practical routes for cryo‐electron tomography and volume imaging in plant structural biology.

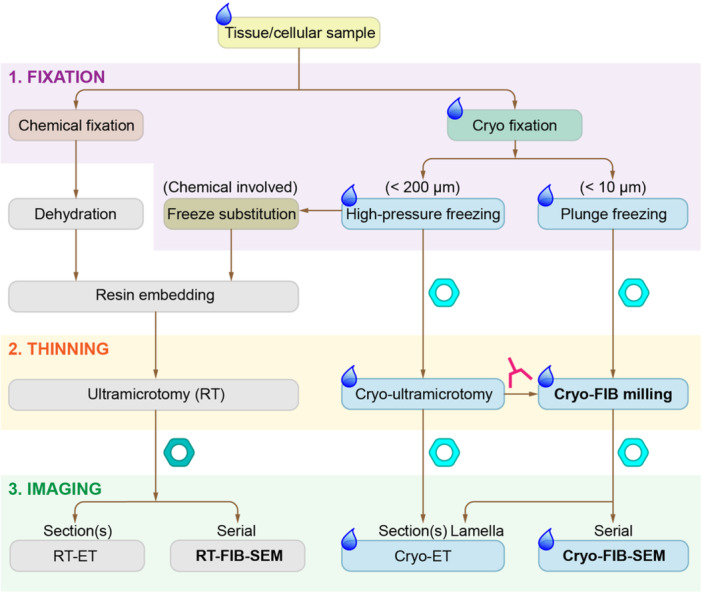

## INTRODUCTION

Cellular compartmentalization facilitates protein trafficking and functions (Alberts et al., 2002). While the spatial organization and behavior of the subcellular compartments reflect their dynamics (Dehmelt and Bastiaens, 2010), their structure and architecture are also important for us to understand the ongoing biological processes among these compartments (Kang et al., 2021). Visualization of subcellular structures is, therefore, expected to reveal native three‐dimensional (3D) information. Most of the subcellular structures (e.g., membranous organelles) can be scaled by microns (Siedlik et al., 2021). As for the finer structures such as ribosome, vesicle coating complex, and components of membrane microdomains, the scale goes down further to the sub‐nanometer level (Paraan et al., 2020; Erdmann et al., 2021; Lu et al., 2024), which is generally beyond the capability of light microscopies. The short‐wavelength nature of the electron beam makes it an ideal illumination source for visualization of biological samples at a scale ranging from cells to macromolecules *in situ* (Chandler and Roberson, 2009; Koning et al., 2018). Therefore, electron microscopy (EM)‐derived imaging techniques, which we refer mainly to scanning electron microscopy (SEM) and transmission electron microscopy (TEM), provide good chances to observe subcellular structures contextually, which will support and complement the dynamic information revealed by live‐cell imaging. The interaction of electrons with the imaged material generates different kinds of signals. The SEM micrograph usually reflects surface information of the imaged objects by the detection of secondary electrons or backscattered electrons, while the TEM micrograph reflects the internal structures of the sample by the detection of electrons transmitted through the sample (Kim et al., 2021).

**Figure 1:**
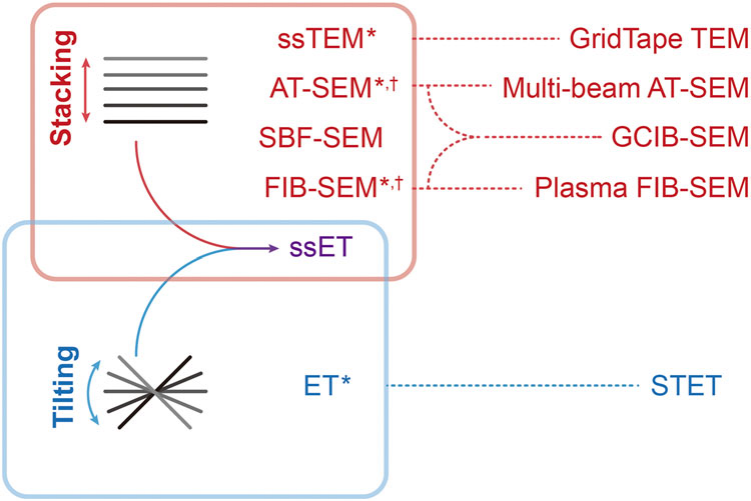
Two ways of generating 3D volumes through electron microscopy (EM) imaging with different modalities under room‐temperature imaging conditions There are two ways of generating 3D volumes through EM imaging. One is stacking serial 2D images taken in the same direction using either scanning electron microscopy (SEM) or transmission electron microscopy (TEM). The other is back‐projecting series of projection images taken by TEM at various angles, which requires sample tilting. The stacking methods include serial section TEM (ssTEM), array tomography SEM (AT‐SEM), serial block face SEM (SBF‐SEM), and focused ion beam SEM (FIB‐SEM). The tilting method includes electron tomography (ET). Additionally, there is a method that combines both the stacking way and the tilting way called serial section ET (ssET). Asterisks indicate that the method has other modalities. GridTape TEM is a variety of ssTEM, which facilitates high‐throughput TEM imaging. Multi‐beam AT‐SEM is a variety of AT‐SEM, where the electron beam is split into multiple parallel beamlets to increase the imaging throughput. Plasma FIB‐SEM (pFIB‐SEM) is a variety of FIB‐SEM, where the focused ion beam is generated from a plasma ion source rather than a gallium ion source. Plasma FIB‐SEM has an advantage in milling large sample areas. Scanning transmission electron tomography (STET) uses the scanning mode of TEM, which is a variety of ET and specialized at imaging thicker samples or densely stained organelles. Gas cluster ion beam SEM (GCIB‐SEM) is a hybrid method of AT‐SEM and FIB‐SEM (marked with daggers), which improves the sectioning reliability as well as the Z‐axis resolution of the AT‐SEM. For a glossary of terms involved in this study, please refer to Table S1.

There are two basic ways to obtain 3D volumes through EM imaging: One is by stacking serial 2D images taken in a same direction using TEM (stacking of 2D projection images of serial sections) or SEM (stacking of 2D scanning images of serial slices or sections), the other is by reconstruction of a series of projection images taken at various tilting angles using TEM (Figure 1). Stacking serial 2D images is more intuitive, including four major methods: (1) serial section TEM (ssTEM), (2) array tomography SEM (AT‐SEM), (3) serial block face SEM (SBF‐SEM), and (4) focused ion beam SEM (FIB‐SEM). The first method generates a 3D volume by stacking 2D TEM projection images and has long been employed to reconstruct 3D volumes from cells and tissues (Birch‐Andersen, 1955; Harris et al., 2006; Frías‐Anaya et al., 2021; Yamane et al., 2022). A variant, GridTape TEM was developed to facilitate the automated high‐throughput TEM imaging (Graham et al., 2019; Phelps et al., 2021). The other three methods are based on SEM imaging. In AT‐SEM, the target sample is first sectioned serially, and the resulting sections are collected on a silicon wafer or a cover slide for SEM imaging. This arrangement facilitates multiple rounds of SEM imaging as required (Micheva et al., 2024). Several variants of AT‐SEM, such as SEM with automated tape‐collecting ultramicrotome (ATUM) and multi‐beam AT‐SEM, were also developed to increase the throughput and efficiency of section and data collection (Hayworth et al., 2014; Eberle and Zeidler, 2018). In SBF‐SEM, the process of mechanical sectioning and imaging is iteratively performed (Denk and Horstmann, 2004). Dual‑beam FIB‑SEM is a microscopy platform that combines an FIB with an SEM in a single instrument. With the advent of dual‐beam FIB‐SEM in biological research, surface ablation of the sample block is accomplished via ion beam milling, giving rise to the technique known as FIB‐SEM (Bushby et al., 2011). The ion beam of FIB‐SEM can be generated from liquid metal ion such as gallium, or from various kinds of plasma gases (Narayan and Subramaniam, 2015; Xu et al., 2017; Sergey et al., 2018). FIB‐SEM avoids the artifacts introduced by the mechanical cutting and largely increases the Z‐axis resolution compared with the other three methods (Kremer et al., 2015; Titze and Genoud, 2016). A variant, gas cluster ion beam SEM (GCIB‐SEM), which can be considered as a hybrid of AT‐SEM and FIB‐SEM, was developed to improve sectioning reliability, increase Z‐axis resolution, and maintain compatibility with multi‐beam AT‐SEM imaging (Hayworth et al., 2020).

Electron tomography (ET) is the technique to obtain 3D volumes by back‐projecting angular projection images. In ET imaging, samples are usually cut into sections with limited thickness (below the mean free path of the electron beam transmission) (Gan and Jensen, 2012). Sections are then imaged under TEM at a wide range of tilting angles (the angular range over which the specimen is incrementally rotated relative to the electron beam), followed by weighted back‐projection of these 2D projection images to generate a 3D volume (McIntosh et al., 2005). Unlike the 3D imaging methods by stacking, where the Z‐axis resolution is determined by section thickness or milling step, ET is powerful in obtaining 3D nanoscale fine structures of organelles in the contextual cellular environment with improved Z‐axis resolution (Wang et al., 2019; Kislinger et al., 2024). In addition, stacking serial reconstructed 3D volumes obtained by ET imaging of consecutive sections can substantially expand the total imaging volume, enabling reconstruction at the whole‐cell level (Cui et al., 2019; Cao et al., 2022; Huang et al., 2022). This can be considered as a hybrid way of stacking and back‐projecting and is often termed serial section ET (ssET) (Kiewisz et al., 2023; Raimondi et al., 2023; Sun and Sui, 2023; Sun et al., 2024). Another modality of ET employs the scanning mode of TEM, known as scanning transmission electron microscopy (STEM). This technique—referred to as STEM‐ET or scanning transmission electron tomography (STET)—is particularly effective for imaging densely stained organelles in relatively thick specimens, albeit with a prolonged acquisition time (Kang, 2016; Ivanov et al., 2019; Liang et al., 2022).

To obtain near‐native ultrastructure, a comprehensive suite of EM‐based technologies has emerged and matured over the past decades, enabling *in situ* 3D subcellular imaging under cryogenic conditions. These include cryo‐FIB milling, cryo‐lift‐out, cryo‐correlative light and electron microscopy (cryo‐CLEM), cryo‐ET, cryo‐STEM‐tomography (CSTET), and cryo‐FIB‐SEM. These technologies are tailored for generating 3D subcellular architectures within native cellular or tissue contexts (Nogales and Mahamid, 2024). The resulting near‐native ultrastructural data often provides new insights that are obscured or lost in chemically processed room‐temperature (RT) samples (Jung et al., 2020; Eskelinen, 2024). The cryo‐FIB milling can be used to prepare cryo‐lamellae for cryo‐ET imaging and to ablate the sample surface in cryo‐FIB‐SEM imaging (Narayan and Subramaniam, 2015; Wagner et al., 2020; Dumoux et al., 2023). Cryo‐lift‐out enables sample extraction/transfer, and cryo‐CLEM facilitates sample targeting during the cryo‐ET sample preparation or cryo‐FIB‐SEM imaging (Kaufmann et al., 2024; Klumpe and Erdmann, 2024). Different types of micromanipulators, including cryo‐gripper and cryo‐needle, have been developed for the cryo‐lift‐out system, with the needle‐type micromanipulator being more flexible, especially during sample releasing (Mahamid et al., 2015; Schaffer et al., 2019; Klumpe et al., 2022). Recently, a new type of cryo‐lift‐out scheme has been developed, where each lift‐out event enables multiple lift‐in operations. This approach significantly enhances the throughput and success rate of lamella preparation from bulk cryogenic specimens (Nguyen et al., 2024; Schiøtz et al., 2024). Cryo‐CLEM enables the *in situ* localization of fluorescent protein‐tagged molecules—such as rare, spatially dispersed, or transiently present targets—by linking fluorescence‐based molecular identity with contextual high‐resolution ultrastructural information (Wilfling et al., 2020; Bieber et al., 2022). Apart from cryogenic imaging conducted by a cryo‐TEM, either cryo‐ET or CSTET, there is a growing trend toward integrating all essential sample preparation and auxiliary functionalities within a single dual‐beam FIB‐SEM platform (Kuba et al., 2021; S. Li et al., 2023b; Smeets et al., 2021; W. Li et al., 2023; Yang et al., 2023).

Most of the methods above have multiple application examples in single cell systems (e.g., yeast, bacteria, algae) or animal cell/tissue systems (e.g., cancer cell lines, neuron cells, cell‐derived matrix, nematode) (M. Li et al., 2021; Peddie et al., 2022; Varsano and Wolf, 2022; Zuber and Lučić, 2022; Hong et al., 2023; McCafferty et al., 2024; Zens et al., 2024). However, due to the intrinsic characteristics (e.g., non‐adhesive growth, presence of large central vacuole) of plant cells, applications of these imaging modalities, particularly under cryogenic conditions, remain limited in vascular plant cells and tissues (Sarkar et al., 2014; Liu et al., 2021; Nicolas et al., 2022; Sanchez Carrillo et al., 2023). Therefore, the development of suitable and accessible workflows is essential to enable broader structural investigations in plant cell biology. Here, based on the commercial dual‐beam FIB‐SEM system, Thermo Fisher Scientific (TFS) Aquilos 2 Cryo‐FIB that integrates the basic RT‐ and cryo‐functions of SEM imaging, ion beam milling, and lift‐out, we tested and developed the three major workflows for 3D subcellular imaging using plant materials.

**Figure 2:**
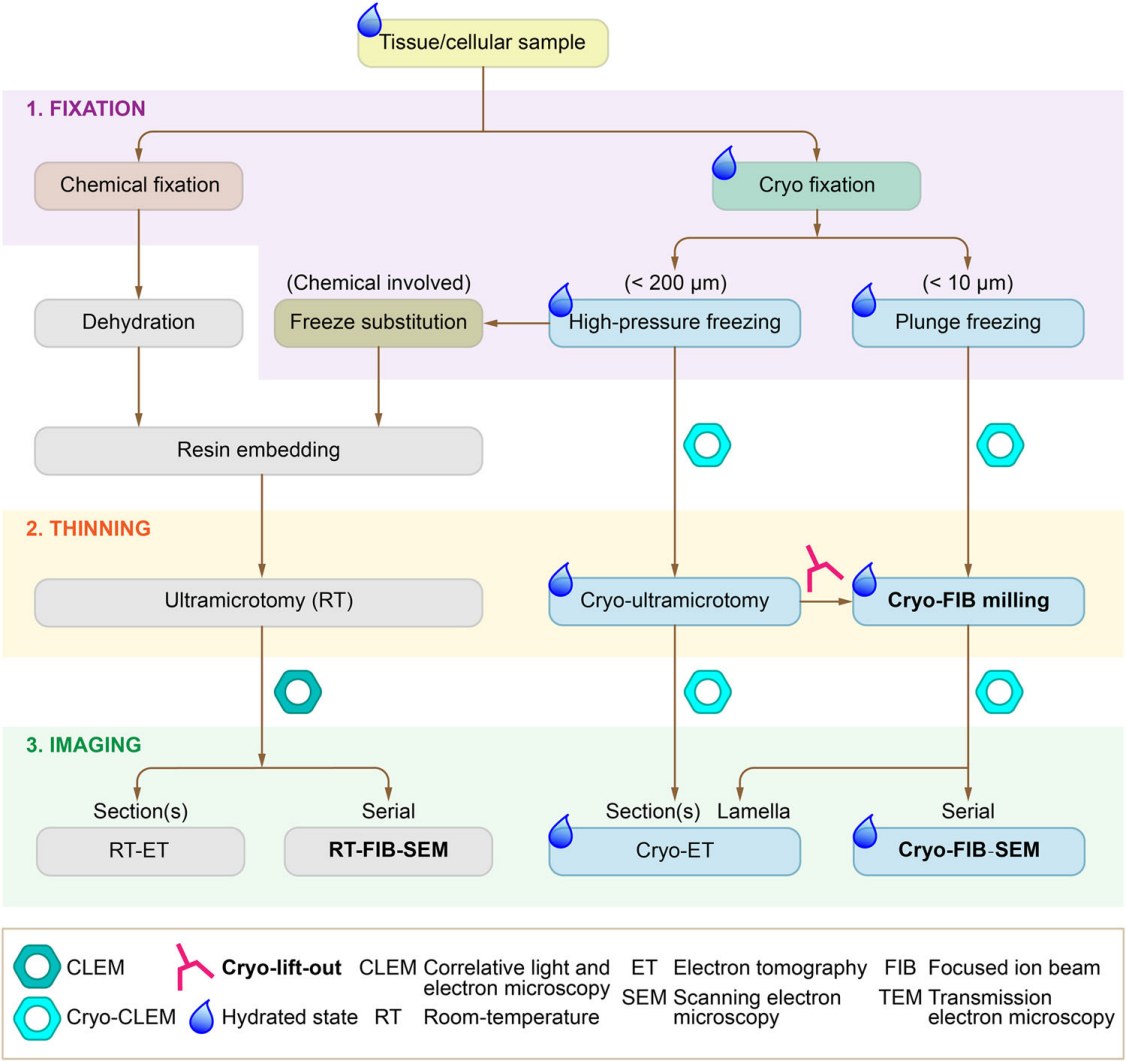
Overview of sample preparation workflows for EM‐based 3D subcellular imaging demonstrated in this study There are normally three steps in sample preparation for EM‐based 3D subcellular imaging: 1. Sample fixation to preserve the tissues/cells as close to the living state as possible (purple box), 2. Sample thinning to trim the fixed samples to a proper volume/thickness for imaging (yellow box), and 3. Imaging to generate 3D data (green box). In the fixation step, cryo‐fixation keeps the materials in a vitreous state, showing superiority over chemical fixation. In cryo‐fixation, high‐pressure freezing (HPF) can cryo‐fix samples up to 200 μm and plunge freezing (PF) can cryo‐fix samples up to 10 μm. In the thinning step, (cryo‐)ultramicrotomy and (cryo‐)FIB milling are two major options. (Cryo‐)Ultramicrotomy achieves thinning by physical cutting using a diamond knife, while (cryo‐)FIB milling achieves thinning by erosion of sample surfaces by a focused ion beam. Optionally, cryo‐lift‐out is used to extract target sample chunks from bulk samples fixed by HPF. In the imaging step, both SEM and TEM can generate subcellular 3D data. SEM usually generates 3D data by stacking serial 2D images taken at a fixed angle. TEM usually generates 3D data by tomography imaging and back‐projecting serial 2D projection images taken at different tilting angles. Cryogenic imaging can preserve the samples in a hydrated state (blue drop labeled), which generates a near‐native structure compared to the other imaging methods. Among all the imaging methods and intermediate steps, (RT‐/cryo‐)FIB‐SEM, cryo‐lift‐out, and cryo‐FIB milling (bold) rely on a dual‐beam FIB‐SEM machine. And CLEM is an optional step bridging molecular identities and contextual subcellular ultrastructure.

Generally, the sample preparation workflows for the major EM‐based 3D subcellular imaging methods (with nanoscale and coarser resolution) involved in this article can be summarized in Figure 2. There are three key steps for each workflow: (1) to fix the cells/tissues at their living state; (2) to open and trim the fixed cells/tissues into smaller/thinner pieces that can be processed under SEM or TEM; and (3) to image the trimmed samples under SEM or TEM. Gray shaded workflows are performed at RT, whereas those shaded in blue correspond to procedures conducted under cryogenic conditions that preserve the cryo‐hydrated state of the specimens. In the fixation step, traditional chemical fixatives penetrate slowly, resulting in inadequate preservation of native cellular architecture. The cryo‐fixation, including high‐pressure freezing (HPF) and immersion or plunge freezing (PF), offers better structural preservation by maintaining the hydrated state of specimens in close approximation to their native cellular conditions. In the thinning step, large‐sized cells/tissues undergo coarse trimming to facilitate subsequent fine thinning, which also allows proper adjustment of sample orientation. For SEM imaging, sample material is progressively removed by FIB‐milling to expose underlying regions for electron beam scanning. For TEM imaging, the roughly trimmed samples are further thinned for electrons to penetrate. This fine thinning is achieved by ultramicrotomy at RT and by cryo‐FIB milling in cryogenic conditions. In the imaging step, RT‐ET can handle either one section or serial sections (as implemented in ssET). Both RT‐ and cryo‐FIB‐SEM capture images of the sequentially generated surfaces. For the cryo‐ET, samples can either be sectioned into serial cryo‐sections using cryo‐ultramicrotomy for serial imaging or prepared as cryo‐lamellae for single lamella imaging.

Pollen development and pollen tube growth are critical for flowering plant fertility and seed setting (Johnson et al., 2019; Hafidh and Honys, 2021). From released microspores to germinating pollen tubes, cells undergo drastic morphological changes and highly dynamic subcellular trafficking (Zhang et al., 2018; Guo and Yang, 2020; Hao et al., 2022). Various live‐cell imaging systems have been applied to the studies of pollen and pollen tube biology (Liu et al., 2022; Ruan et al., 2023). However, the available native subcellular structure remains limited and is outdated (Lancelle and Hepler, 1992; Yamamoto et al., 2003). The tissue‐embedded nature of pollen grains, coupled with the availability of *in vitro* culture systems for pollen tubes, renders them ideal specimens for advancing EM‐based 3D subcellular imaging techniques. Here, using developing anthers and *in vitro*‐cultured pollen tubes, we demonstrated: (1) two strategies for RT‐FIB‐SEM volume imaging, (2) cryo‐lamellae preparation from PF‐ and HPF‐prepared cell/tissue samples for cryo‐ET imaging, and (3) cryo‐FIB‐SEM volume imaging of PF‐ and HPF‐prepared cell/tissue samples. We focused on the illustration of sample preparation workflows in the different use cases and discussed the application of two cryo‐CLEM systems on these samples, providing multiple options as templates for the 3D subcellular imaging of plant cells/tissues based on the commercial Aquilos 2 Cryo‐FIB.

## RESULTS

### RT‐FIB‐SEM volume imaging

#### RT‐FIB‐SEM imaging of developing pollen grains in anther

**Figure 3:**
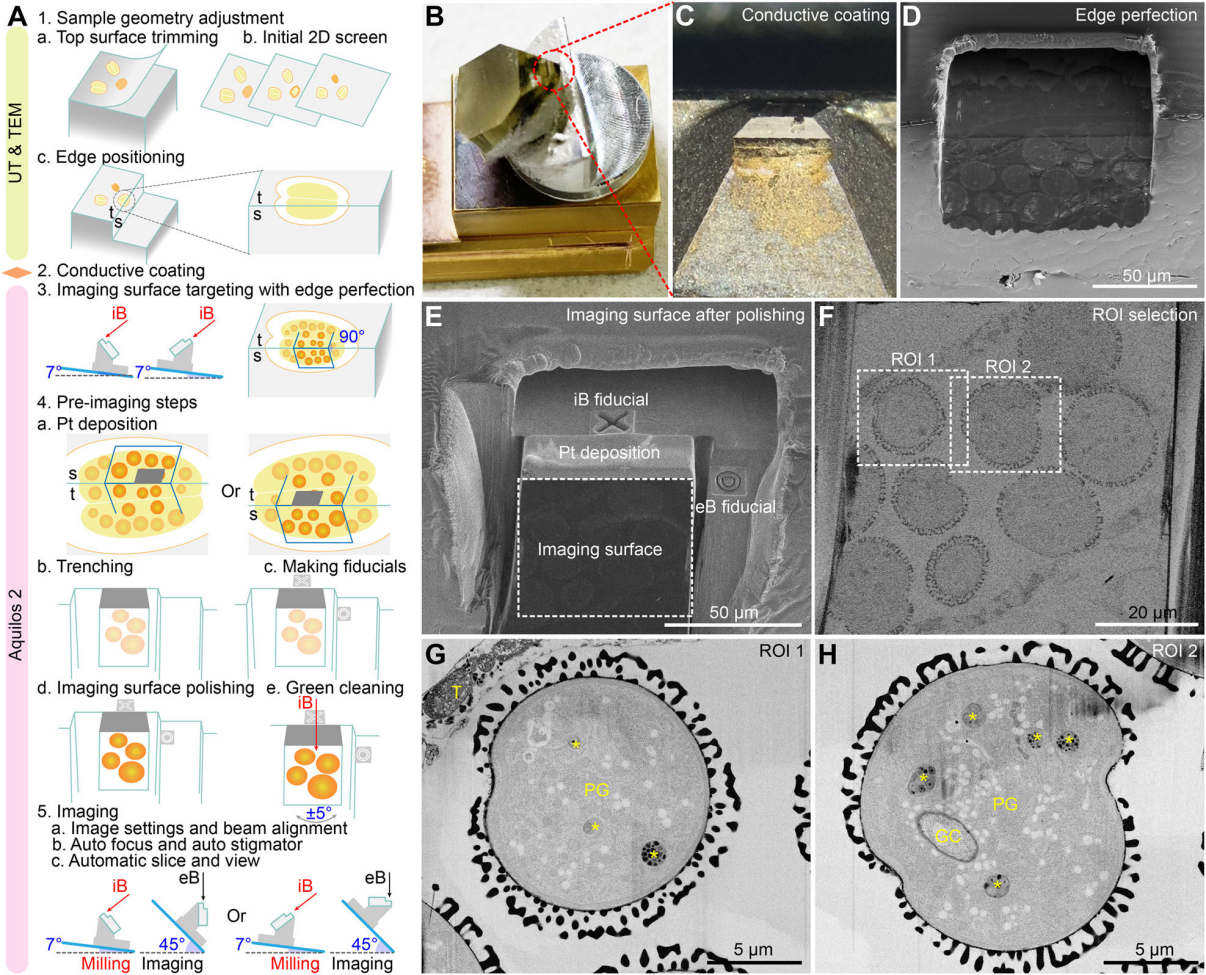
**Perpendicular imaging scheme of RT‐FIB‐SEM using developing** ***Arabidopsis*** **pollen within anther** **(A)** Illustrated workflow of the RT‐FIB‐SEM imaging of the pollen grains from *Arabidopsis* anthers prepared by high‐pressure freezing (HPF) and freeze substitution (FS). Prepared sample blocks are glued on the 45° SEM mount pin and first undergo top surface trimming by ultramicrotomy (UT) (1a) and the initial screening by 2D transmission electron microscopy (TEM) (1b) to make the interested anther tissues exposed, after which the sample block will be further trimmed by ultramicrotomy so that the interested anther is positioned at a newly trimmed edge (1c). The trimmed sample block is then painted with conductive silver paint and coated with conductive metal by a sputter coater (2). After transferring the sample into Aquilos 2 chamber, the imaging area is finely targeted with edge perfection (by polishing the roughly perpendicular planes with an ion beam under different stage positions) (3). A protective organometallic platinum layer is deposited on the milling area perpendicular to the imaging surface with two optional geometries (4a). The area without the Pt layer will be milled away in the trenching step (4b). Two different fiducial markers will be created near the milling area and beside the imaging area for automated milling and imaging, respectively (4c). The imaging surface is fine polished manually (4d) or by the *Green Clean* function of the Auto Slice & View (ASV) software with an angular rocking mill (4e). After proper image settings and beam alignment, the automated FIB‐milling and image collection can be started (5). Electron beam is perpendicular to the imaging surface in both optional geometries (5). eB, electron beam; iB, ion beam; Pt, platinum coating; s, side surface; t, top surface. **(B)** A trimmed sample block on the 45° surface of the SEM mount pin on the brass standard shuttle. **(C)** Enlarged view of the trimmed and conductive metal‐coated sample block. The sample surface turns silver‐gray after the conductive coating. **(D)** SEM view of the exposed area after edge perfection. Multiple pollen grains can be found on both surfaces. **(E)** The polished imaging surface with the surrounding material milled away. The fiducial marker for ion beam milling is near the milling area (area with Pt deposition), and the fiducial marker for electron beam imaging is beside the imaging surface. **(F)** Enlarged view of the imaging surface under backscattered electron detection mode, within which two regions of interest (ROI) are selected for further examination. **(G**, **H)** Representative output high‐resolution SEM images from the selected ROIs. GC, generative cell; PG, pollen grain; T, tapetum. Yellow asterisks indicate plastids. Scale bars, 50 μm in **(D**, **E)**; 20 μm in **(F)**; 5 μm in **(G**, **H)**.

Previously, using the whole‐cell ET/ssET, we analyzed the initial and subsequent cells in the root cortex of *Arabidopsis*, revealing the morphological characteristics and formation mechanisms of vacuoles during early cortex development (Cui et al., 2019). The imaging modality was extended to the investigation of different types of plant cells, including stomatal lineage cells and developing pollen (Cao et al., 2022; Liang et al., 2024). Mature‐staged pollen can reach 20 μm in diameter (i.e., mature pollen grains of *Arabidopsis*). Although ssET covering whole‐cell region allows the atlas view of the organelle morphology and distribution at nanoscale resolution (usually 3–6 nm imaging pixel size), the high contrast between the spiked pollen coat and the cytosol content, the warping of plastic sections, and the increased scattering of the lateral regions caused by tilt geometry during imaging impeded us from obtaining high‐quality whole‐cell tomograms (Figure S1). We, therefore, turned to the RT‐FIB‐SEM imaging to obtain whole‐cell volumes of *Arabidopsis* pollen grains within anthers in late developmental stages (Figure 3).

Sample preparation of the RT‐FIB‐SEM imaging was similar to that of the RT‐ET (Figure 2). Key steps after the successful sample embedding are illustrated in Figure 3A, including: (1) Sample geometry adjustment, (2) Conductive coating, (3) Imaging surface targeting with edge perfection, (4) Pre‐imaging steps, and (5) Imaging. Sample geometry adjustment was achieved by ultramicrotomy and intermittent examination of the ultrathin sections trimmed off from the top and side surfaces (Figure 3A1). The aim of the step is to roughly target the interested tissue to the edge of the block, where the trimmed top surface (labeled by “t” in Figure 3A) is roughly perpendicular to the trimmed side surface (labeled by “s” in Figure 3A). Conductive coating is the step to increase the conductivity of the resin block surface before transferring it into the dual‐beam microscope (Figure 3A2, C). We used low profile 45°/90° pin stub to facilitate the relative geometry of the imaging and milling surfaces to the corresponding beams. Manual attachment of the sample block to the pin stub, ultramicrotomy trimming, and sample transfer to the dual‐beam microscope chamber; each of these steps may affect the relative angles of the two surfaces (t and s) to the dual beams. To make the sample‐containing edge of the ultramicrotomy‐trimmed block at a perfect right angle, as well as to align the t and s surfaces to the dual beams, the top and side surfaces near the edge were milled by an ion beam at two different stage geometries (Figure 3A3), thus creating two new surfaces that are strictly orthogonal to each other (Figure 3D). The two newly generated surfaces were then briefly examined by SEM to determine which would serve as the milling surface and which would serve as the imaging surface (Figure 3A4a). Each of the surfaces can serve as a milling or imaging surface, which depends on the distribution of the interested cells/features. Upon selection of the imaging surface, a protective platinum (Pt) layer was deposited on the corresponding area of the milling surface by beam‐induced Pt deposition from organometallic precursor using the gas injection system (GIS), to protect the surface of the milling area from ion beam erosion (Figure 3A4a, E). To avoid the continuous redeposition of the milled material and the charging effects exacerbated by the repetitive milling, material surrounding the milling/imaging area should be cleared in advance (Figure 3A4b, E).

The FIB‐SEM is the iteration of FIB‐milling to create new serial imaging surfaces and the SEM imaging of the newly created sample surfaces. The automation of this iteration is achieved by Auto Slice & View (ASV), the supportive software of Aquilos 2 Cryo‐FIB. To facilitate the recognition of the same milling and imaging area in each iteration cycle by the ASV, two different fiducial patterns (the cross pattern and the circle pattern) were drawn near the milling and imaging area, respectively (Figure 3A4c, E). Optionally, before the establishment of the automation task, the imaging surface, with milled material redeposited during the trenching step, can be manually polished to generate a perfect initial surface for imaging (Figure 3A4d, E). There is also an exclusive feature of ASV for this imaging workflow (SEM scanning at 90° to the imaging surface using low profile 45°/90° pin stub) called rocking mill, which is designed to avoid milling curtain during the ASV‐controlled polishing of the imaging surface (*Green Clean* function in the ASV, Figure 3A4e) before region selection and SEM scanning parameters settings.

The automation task can be set following the workflow tabs within the ASV. Key settings such as regions of interest (ROI), electron scan resolution, electron scan dwell time, areas/conditions/frequencies of auto‐focus and auto‐stigmator, milling step, milling depth of the ion beam, and the milling thickness in total can be defined in the corresponding tabs of the ASV software (Figure 3A5). ROI is the area of serial image data collection (Figure 3F). Electron scan resolution depends on the pixel numbers in the width and height of the ROI selection image (Figure 3F) when the ROI selection image size is fixed. Electron scan dwell time is the time that the electron beam remains in each spot/pixel. Longer dwell time increases the signal out of the electron‐sample interaction but brings more electron damage to the sample. Auto‐focus and auto‐stigmator areas are usually set in the same horizontal line in the middle of each ROI, with some high‐contrast features existing throughout the ASV FIB‐SEM task. The milling step is the slice thickness in each milling‐imaging iteration cycle, which reflects Z axis resolution of the output serial image dataset. The milling depth of the ion beam determines the smooth imaging surface area that we can obtain from each milling action. And the milling thickness in total is the Z depth of the whole imaging volume, which should fall within the protective Pt deposition area. These settings are not necessarily input at a time in the ASV, and one needs to switch back and forth between the microscope controlling software (xT user interface, xT UI) and the ASV to make sure the detector and *Use Case* mode are suitable and to fine‐tune the beam geometry in *Direct Adjustments* right before SEM scanning settings in the ASV. Multiple ROIs can be selected by setting different sets of *SEM Image*s (Figure 3F–H). This function is useful to obtain multiple examples from one automation task when the imaging surface is large enough. There were chances of new pollen grains emerging when the FIB‐milling went deeper. Optionally, the original ASV program can be stopped and replaced with a new task focusing on other newly emerged pollen grains to generate multiple datasets.

#### RT‐FIB‐SEM imaging of *in vitro*‐cultured pollen tube

Previously, we used RT‐ET to characterize the tip‐vesicles in high‐pressure‐frozen and freeze‐substituted pollen tubes from lily, revealing the presence of various vesicles such as electron‐translucent secretory vesicles, electron‐dense vesicles, mini vesicles, and extracellular vesicles (Liu et al., 2021). For the cross‐validation across multiple species, we expanded our investigation into other commonly used model species in pollen tube studies, such as tobacco. During our routine ssET analysis of tobacco pollen tube tip‐vesicles, we found the distinct lumen densities between the two halves of the same vesicle embedded within two adjacent sections (yellow arrowheads in Figure S2). From the XZ or YZ view/tomographic slice of the joined tomogram volume, some of the half‐vesicles with light electron density exhibit a cone‐shaped “hollow” region (white arrowheads in Figure S2). This observation is reminiscent of the missing wedge artifact in ET (Koning et al., 2018). However, the fact that the triangle “hollows” only exist on the surfaces on the same side of the serial sections indicates other reason(s) for this observation, for example, the surface material loss during the mechanical cutting in ultramicrotomy. To tackle this problem, we turn to RT‐FIB‐SEM imaging.

**Figure 4:**
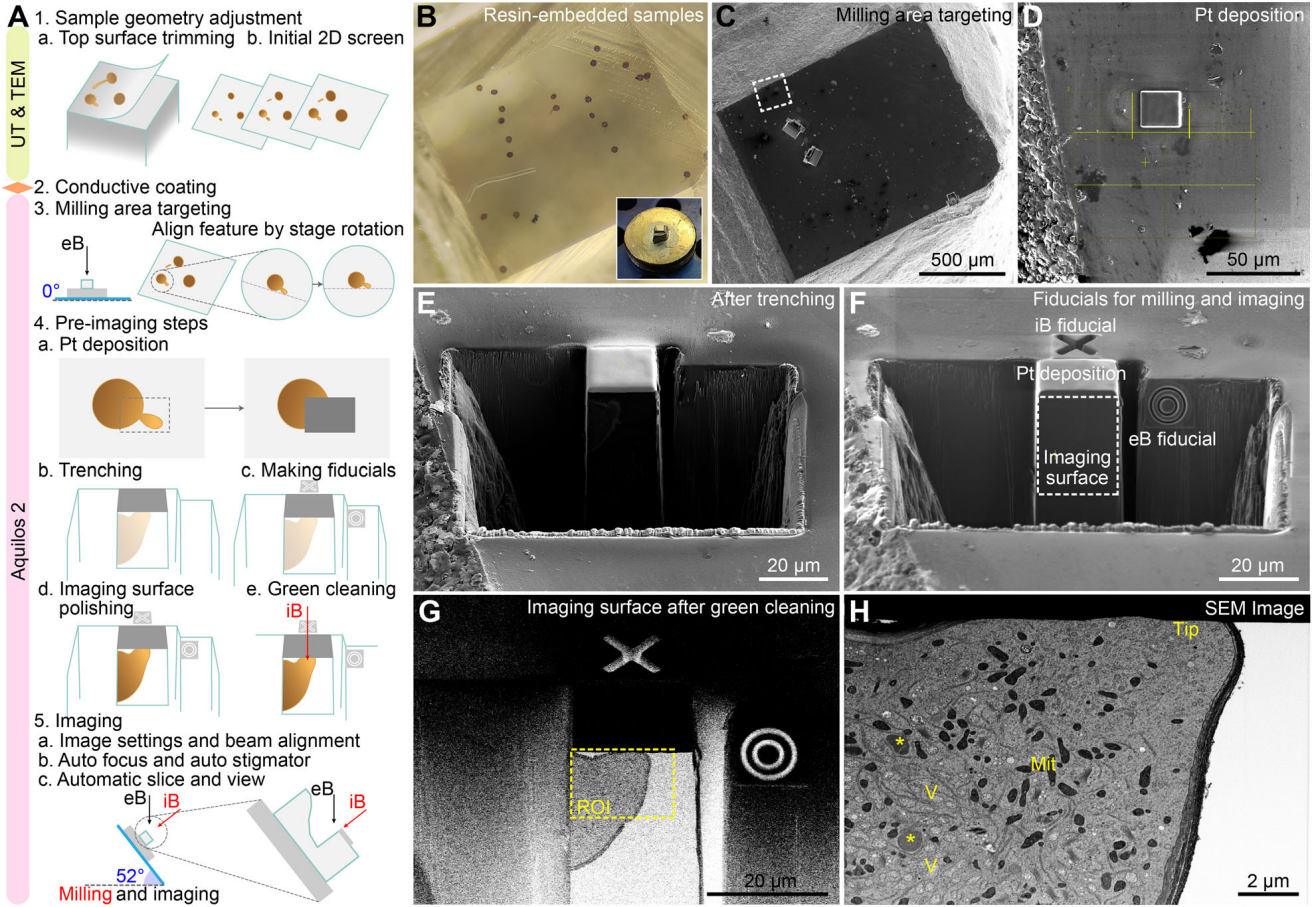
**Tilted imaging scheme of RT‐FIB‐SEM using** ***in vitro*** **‐cultured tobacco pollen tubes** **(A)** Illustrated workflow of the RT‐FIB‐SEM imaging of the *in vitro*‐cultured tobacco pollen tubes prepared by high‐pressure freezing (HPF) and freeze substitution (FS). Sample block is glued on the flat SEM pin stub mount. Sample block surface is trimmed by ultramicrotomy (UT) (1a), followed by initial screening using 2D transmission electron microscopy (TEM) (1b) to pinpoint the interested pollen tubes. The trimmed sample block is then painted with conductive silver paint and coated with conductive metal by a sputter coater (2). After transferring the sample into the Aquilos 2 chamber, fine target of the cell of interest according to the 2D TEM screening images and the SEM scanning view of the block surface. The target cell will be aligned to the designated angle by stage rotation (3). A protective organometallic platinum (Pt) layer is deposited on the milling area covering the estimated imaging area (4a). And the area without the Pt layer will be milled away in the trenching step (4b). Two different fiducial markers will be created near the milling area and beside the imaging area for automated milling and imaging, respectively (4c). The imaging surface is fine‐polished manually (4d) or by the *Green Clean* function of the Auto Slice & View (ASV) software (4e). After proper image settings and beam alignment, the automated FIB‐milling and image collection can be started at the fixed stage tilt of 52°, with the electron beam and the imaging surface remaining at a 52° angle (5). eB, electron beam; iB, ion beam; Pt, platinum coating. **(B)** A trimmed sample block surface under a stereomicroscope. Inlet shows the same block mounted on the flat SEM pin stub mount, painted with conductive silver paint, and coated with conductive metal. **(C)** SEM overview of the sample block surface. **(D)** Enlarged view of the dashed box area in **(C)**, with the milling area coated with a protective Pt layer. Trenching areas are indicated by yellow boxed patterns. **(E)** SEM view of the coated milling area after the trenching step. **(F)** The fiducial marker for ion beam milling is made near the milling area (area with Pt deposition), and the fiducial marker for electron beam imaging is made beside the imaging surface. **(G)** Imaging surface overview after green cleaning under backscattered electron detection mode. A region of interest (ROI) covering the tip region of the pollen tube is selected for high‐resolution imaging. **(H)** A representative output high‐resolution SEM image of the selected ROI. Mit, mitochondria; Tip, pollen tube tip region; V, vacuole. Asterisks indicate plastids. Scale bars, 500 μm in **(C)**; 50 μm in **(D)**; 20 μm in **(E–G)**; 2 μm in **(H)**.

Sample preparation was similar to that of the RT‐ET (Figure 2). Unlike the developing pollen grains wrapped within the pollen sacs of anther, the tobacco pollen tubes embedded in the resin were dispersed. Therefore, we adopted a different working scheme of RT‐FIB‐SEM, which is more friendly to screen, target, and orient the cells under SEM. Key steps after the successful sample embedding are illustrated in Figure 4A, including: (1) Sample geometry adjustment, (2) Conductive coating, (3) Milling area targeting, (4) Pre‐imaging steps, and (5) Imaging. The sample geometry adjustment for this scheme is not as complex as the previous one for RT‐FIB‐SEM imaging of developing pollen. No orthogonal surfaces were created in this step, yet ultramicrotomy and intermittent examination (by 2D TEM) of the ultrathin sections trimmed off from the top surface helped the screening of well‐prepared pollen tubes and their corresponding tip region (Figure 4A1). Another important goal of this step is to make cells exposed on the trimmed surface so that they are detectable under the SEM. Similar to the RT‐FIB‐SEM imaging of developing pollen, conductive coating was conducted before transferring the sample block into the dual‐beam microscope (Figure 4A2, B). We used flat stub pins in this workflow. After sample transfer, the interested cell was located under SEM (Figure 4A3, C) with the aid of block surface shape, stereo microscopic image of the block surface (Figure 4B), and the 2D TEM micrographs of the sections cut for screening in the first step. The interested cell was then aligned horizontally, which depends on the interested biological feature and the desired angle of view (Figure 4A3). An estimated area of milling was then coated with a protective Pt layer (Figure 4A4a, D), and material surrounding the milling/imaging area was cleared in advance by FIB‐milling (Figure 4A4b, E).

Similar to the RT‐FIB‐SEM imaging of developing pollen, two different fiducial patterns (the cross pattern and the circle pattern) were drawn near the milling and imaging area, respectively, to facilitate the recognition of the same milling and imaging area in each iteration cycle by the ASV software (Figure 4A4c, F). The imaging surface was polished manually (Figure 4A4d) or by the *Green Clean* function of the ASV (Figure 4A4e). Different from the working scheme for imaging developing pollen, the rocking mill cannot be applied in the *Green Clean* function here. Therefore, the milling depth of the green cleaning should be large enough to create sufficient smooth area for the imaging of the interested features. By gradually discarding the surface material from the imaging surface, the *Green Clean* function can also be used to locate the intended initiating section for real data collection to save time. Other key settings, as listed above in the RT‐FIB‐SEM imaging of developing pollen, were then input to build the automation task in the ASV interface (Figure 4A5). We selected one ROI covering the estimated tip region of the pollen tube for SEM data collection (Figure 4G, H). If one only focuses on specific subcellular regions, for example, the tube apex region, the connection region between the emerging pollen tube and the pollen grain, or the cell wall regions, indeed, multiple restricted ROIs can be selected with higher electron scan resolution depending on the biological questions.

### Cryo‐lamellae preparation for cryo‐ET imaging

Sample preparations for RT‐ET and RT‐FIB‐SEM imaging share similar steps (Figure 2). They both involve chemical substitution and resin embedding. These steps may introduce artifacts and cannot be bypassed if the imaging step is conducted at RT. There is a huge gap between the cryo‐fixation temperature (the temperature of liquid nitrogen) and the RT. To preserve the cellular ultrastructure, temperature ramping and resin infiltration have to be slow and stepwise (Kang, 2010). Samples finally become dehydrated after these steps. Any abrupt temperature change, inappropriate freeze‐substitution cocktail combination, or insufficient incubation time may adversely affect the structure preservation. Heavy metal staining also brings uncertainties to the repeatability of the final ultrastructure. Compared to the RT‐imaging modalities, cryo‐imaging methods allow better observation of near‐native ultrastructure within hydrated cells without heavy metal staining, albeit with the restricted rounds of imaging and sample thickness (imaging volume). Cryo‐ET suits more in the cases below: (1) Structures that can only be observed under near‐native state or in hydrated cells, or structures sensitive to RT‐sample preparation chemicals (e.g., cryo‐ET complements the poor visualization of actin filaments in RT‐imaging samples) (Lancelle and Hepler, 1991; Otegui et al., 2001; Schneider and Jasnin, 2022); (2) more accurate identification of subcellular structure is required to further confirm the ambiguous structures obtained by RT‐imaging methods (e.g., different coated‐vesicles can be visually identified in the tomograms without any ambiguity; Bax accumulation adjacent to mitochondria was better identified under cryo‐ET) (Donohoe et al., 2007; Bykov et al., 2017; Ader et al., 2019; Borgeaud et al., 2024); (3) *in situ* macromolecule structures are expected (Briggs, 2013; Turoňová and Wan, 2024). Although cryo‐ultramicrotomy can generate cryo‐section ribbons thin enough for cryo‐TEM imaging, the mechanical cutting introduces many artifacts on the section (chatter, crevasses, fractures, and knife marks) and causes sample compression in the cutting direction (Al‐Amoudi et al., 2005; Hylton and Swulius, 2021). FIB‐milling function under cryogenic conditions generates smoother cryo‐lamellae for cryo‐ET imaging and allows the sample thinning for cryo‐FIB‐SEM imaging.

#### Cryo‐lamellae preparation from suspension culture of *Arabidopsis* pollen tubes

Small suspension culture cells (< 10 μm) are friendly to PF. Early applications of cryo‐FIB milling to biological samples utilized suspension cell culture systems (e.g., yeast, algae) and adherent cell systems (e.g., neurons). Yeast and algae cells exhibit small clustered “hills” on grids after PF (Engel et al., 2015; Bieber et al., 2021). Mammalian adherent cells are relatively flat and exhibit sheet‐like spreading on grids (Guo et al., 2018). These shapes are rather regular and amenable to FIB‐milling. *In vitro* culture is also frequently used in pollen tube studies. Although the total size of the pollen tube together with the connected pollen grain exceeds the ideal freezing depth of PF, the pollen tube part of small‐sized species (e.g., pollen tube diameter of *Arabidopsis* is around 10 μm), the tip region and peripheral part of the pollen tube can be well cryo‐fixed. Our interest lies in the tip‐vesicles of growing pollen tubes (Liu et al., 2021, 2025). Therefore, to obtain near‐native ultrastructure, we followed the routine cryo‐FIB milling methods to prepare cryo‐lamellae from *in vitro*‐cultured *Arabidopsis* pollen tubes.

**Figure 5:**
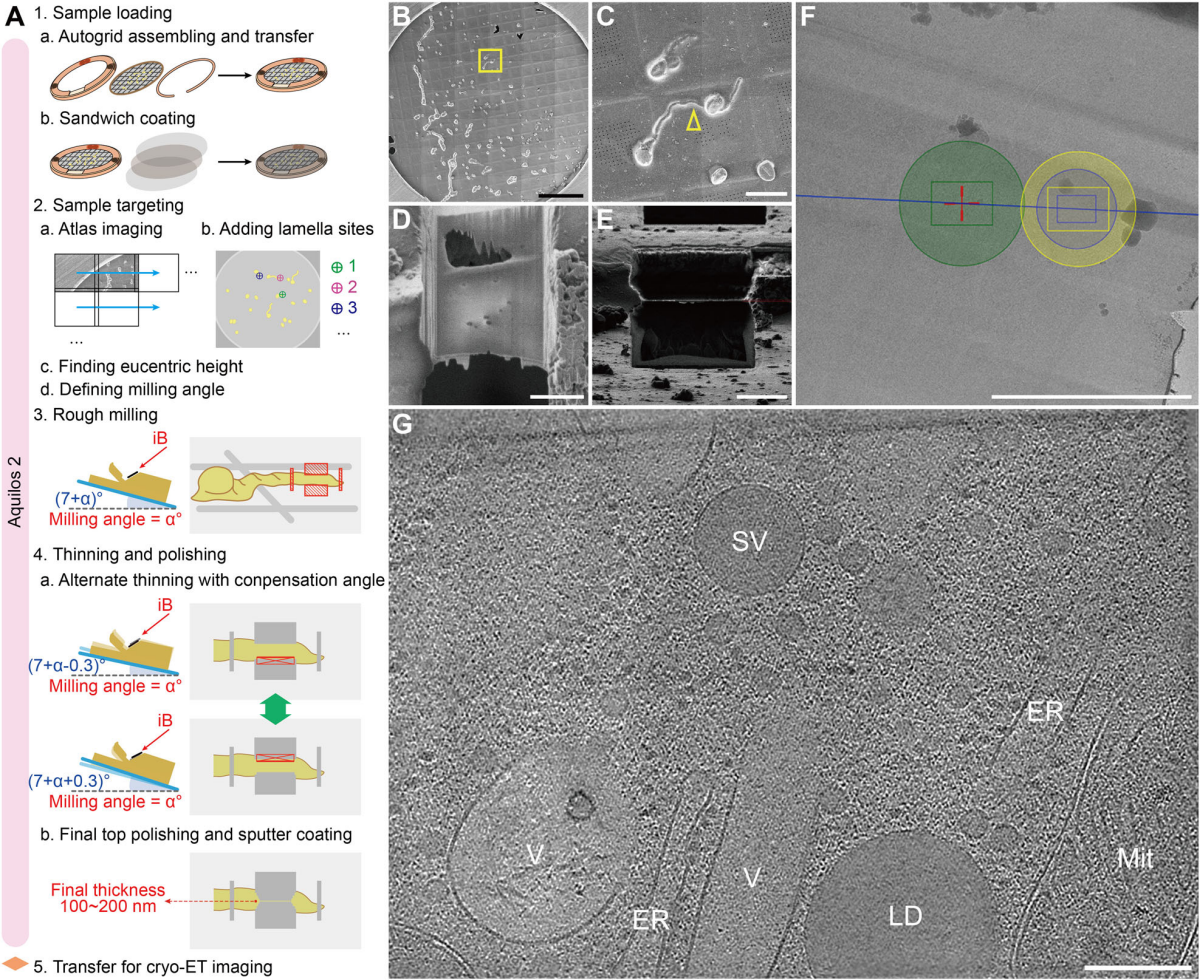
**Cryo‐ET imaging of a cryo‐FIB‐milled lamella from plunge‐frozen** ***Arabidopsis*** **pollen tubes** **(A)** Illustrated workflow of the cryo‐lamella preparation for cryo‐ET imaging. A plunge‐frozen grid bearing *in vitro*‐cultured *Arabidopsis* pollen tubes is first assembled into Autogrid (1a). After being transferred into Aquilos 2 chamber, the Autogrid is subjected to sandwich coating, including a first sputter coating of conductive platinum, a dense protective layer of organometallic platinum, and a second conductive sputter coating (1b). The coated Autogrid is then tile‐imaged with MAPS software to obtain a grid atlas at a 180° scan rotation (2a), during which one can add target lamella sites in the software interface (2b). Coordinates of the added lamella sites are updated with eucentric height and milling angle if one chooses to navigate the stage with MAPS. Alternatively, the eucentric height and milling angle can be determined manually in the xT UI microscopy control software (2c, d). The target cell is opened in the rough milling step (3), followed by iterative thinning of the bottom and top sides of the lamella chunk using a 0.3° offset angle to compensate for the faster milling rate at the lamella front edge compared to the tail end (4a). Make sure that final polishing is applied to the top side of the lamella and perform an additional short sputter coating to increase lamella conductivity for subsequent cryo‐TEM imaging after all lamella sites have been processed to their final thickness, typically 100–200 nm (4b). The Autogrid will be ready for cryo‐ET imaging after proper transfer from the Aquilos 2 to a cryo‐TEM. **(B)** The tiled atlas view of a sandwich‐coated grid obtained by MAPS. **(C)** Enlarged view of the box area in **(B)** showing a target lamella site (arrowhead) before cryo‐FIB milling. **(D)** Scanning electron micrograph of the final lamella generated from the target site in **(C)**. **(E)** Ion beam view of the final lamella generated from the target site in **(C)**. **(F)** Search view of the same lamella shown in **(D**, **E)** under cryo‐TEM. The blue central line indicates the tilting axis. The green, yellow, and blue circles indicate the beam spots of exposure, tracking, and focus steps respectively, with the boxes inside showing the corresponding camera detection areas. **(G)** A tomographic slice of the exposure area in **(F)**. ER, endoplasmic reticulum; LD, lipid droplet; Mit, mitochondrion; SV, secretory vesicle; V, vacuole. Scale bars, 500 μm in **(B)**; 50 μm in **(C)**; 5 μm in **(D–F)**; 200 nm in **(G)**.

Steps after successful sample fixation are illustrated in Figure 5A, including: (1) Sample loading, (2) Sample targeting, (3) Rough milling, (4) Thinning and polishing, and (5) Transfer and cryo‐ET imaging. Autogrid for FIB‐milling includes an O‐ring and a C‐ring. Clipping of bare grid into the Autogrid assembly increases the resistance to deformation during grid transfer by tweezers (Figure 5A1a). The O‐ring has an opening slope (milling slot) for FIB‐milling at a low milling angle (angle between the ion beam and the grid plane, usually ranging from 6° to 15°). Markers were labeled on the O‐ring prior to grid clipping for better recognition of the Autogrid direction (Figure 5A1a). The assembled Autogrid was then transferred into the dual‐beam microscope chamber, followed by a three‐layer sandwich coating, with a thick protective Pt layer (organometallic platinum) in between two thin Pt layers (platinum metal) (Figure 5A1b). The thick organometallic platinum coating, conducted by GIS, protects the leading edge (the front surface of the cell facing the ion beam) from uneven ion beam erosion during the FIB‐milling, thus avoiding the curtaining artifact (Bisson et al., 2021). The thickness of the protective Pt layer is related to multiple factors, such as the sufficiency of the organometallic platinum source, the distance between the GIS needle tip and the grid surface, and the coating time. Usually, the GIS needle position was tuned to a fixed position during microscope installation, and the default *deposition position* of the stage was stored in the xT UI. With these geometries settled, coating time was generally between 30 s and 1.5 min. But this should be tested first to obtain an empirical value for each dual‐beam microscope. Too thick of the GIS coating will lead to the breaking of the grid film. The thin Pt metal coating, conducted by a platinum sputter coater, increases the conductivity of the sample surface, thus reducing the charging effects and improving the SEM imaging quality. After the first layer of thin Pt metal coating, one can search different areas of the grid to gain a fast look at the sample quality (e.g., cell density and distribution). If the grid is not good enough for further FIB‐milling, the other two layers of the coating can be omitted to save time for examination of the next grid. Each sample loading can handle two grids using the Autogrid shuttle.

An auxiliary software, MAPS, was used to gain an atlas view of the grid by tiled imaging with the desired atlas area, tile numbers, and imaging resolution (Figure 5A2a, B). During the tiled imaging process, interested milling targets can be added as *lamella site*s recorded in the MAPS project (Figure 5A2b, C). For each lamella site, to make sure the milling target is centered at the electron beam (eB) view and the ion beam (iB) view, the milling target needs to be located at its eucentric height where eB and iB converge at the milling target (Figure 5A2c). And the milling angle can be set as the minimum angle without the rim of the O‐ring obscuring the iB view of the milling target (Figure 5A2d). Optionally, the milling angle can be set uniformly for all the lamella sites on one grid (e.g., 8°). These two steps can be achieved under the guidance of MAPS. Based on the geometry of the stage and the dual‐beam system, the iB is parallel with the grid plane when the stage tilt is 7°, and a 45° Pre‐Tilt Autogrid shuttle is applied. Therefore, when the milling angle is set as α°, the stage tilt should be (7 + α)° (Figure 5A3). These two steps can be achieved under the guidance of MAPS. Optionally, the lamella sites can also be imported to another software, AutoTEM Cryo, for automation. In the rough milling, two parallel *Rectangle Pattern*s were drawn with a vertical spacing of 4 μm, aligned to the expected lamella position of the cell. The horizontal edges of the patterns facing the cell should be the directions for iB approaching. Therefore, the *Scan Direction* of the top *Rectangle Pattern* should be set *Top To Bottom,* and the *Scan Direction* of the bottom *Rectangle Pattern* should be set *Bottom To Top*. This rule also suits the following steps of milling. Optionally, two vertical relief cuts can be conducted several microns away from both ends of the expected lamella fabrication area to buffer the tension transferred from the other cell portions or surrounding grid film (Wolff et al., 2019) (Figure 5A3). The following thinning steps were conducted by milling off the material alternately from the bottom and top of the expected lamella fabrication area with the *Cleaning Cross Section Pattern* (Figure 5A4a), until the thickness of the lamella was reduced to 300–400 nm. It is good for the lamella stability to conduct the thinning FIB‐milling with paired bottom and top milling. Patterns for each paired bottom and top milling were set with the same width. And the pattern width was reduced (e.g., by 0.5 μm) in each round of bottom and top milling, forming steps between the lamella area and the remaining cell portions under iB view. To compensate for the lower milling efficiency at the tail side (downstream of the iB) of the lamella compared to that of the front side (upstream of the iB), a compensation angle of 0.3° was applied to each thinning FIB‐milling pattern (Figure 5A4a). When there are multiple lamella sites, rough milling of all sites should be completed before the final polishing step. The final polishing of the rough‐milled lamellae was then conducted with low milling current (10 pA or 30 pA), proceeding sequentially from the iB‐upstream lamella to the iB‐downstream lamella. We used paired bottom and top milling in the polishing step and placed the last polishing cut at the top of the lamellae (Figure 5A4b). The final lamellae thickness fell between 100 nm and 200 nm (Figure 5A4b, E). It helps in obtaining a lamella close to 100 nm to manually monitor the progress of the lamella top polishing by frequent SEM scanning (e.g., every 5 s) until the front or tail of the lamella starts to lose larger area(s) of material. After all lamellae were polished to satisfaction, grids were briefly coated with a final thin layer of Pt metal coating for better TEM imaging (Figure 5A4b, F). Near‐native subcellular structures such as secretory vesicle, tubular vacuole with intraluminal vesicles, rough endoplasmic reticulum, lipid droplet, and mitochondrion can be identified in the reconstructed cryo‐tomogram (Figure 5G).

#### Cryo‐lamellae preparation from high‐pressure‐frozen *Arabidopsis* anthers by serialized on‐grid lift‐in sectioning for tomography (SOLIST)

Not all types of plant cells have well‐established *in vitro* culture systems available. *In situ* and *in vivo* studies are of great importance in providing definitive evidence for many biological questions. This necessitates the application of cryo‐imaging techniques to cells within plant tissues or even whole organs. The dimensions of multicellular plant tissues typically range from tens to hundreds of micrometers (Fry, 2016). For example, the diameters of *Arabidopsis* root tip and shoot apical meristem are around 100 μm. And *Arabidopsis* anthers can reach their mature length of 350–400 μm at stage 12 of floral development (right before pollination) (Alvarez‐Buylla et al., 2010). Therefore, HPF is required for cryo‐fixation. To obtain *in situ* structures of the vacuoles in developing *Arabidopsis* pollen grains, we used the needle system (EasyLift) of cryo‐lift‐out to extract sample chunks containing pollen grains from the bulk high‐pressure‐frozen anthers for lamellae preparation.

**Figure 6:**
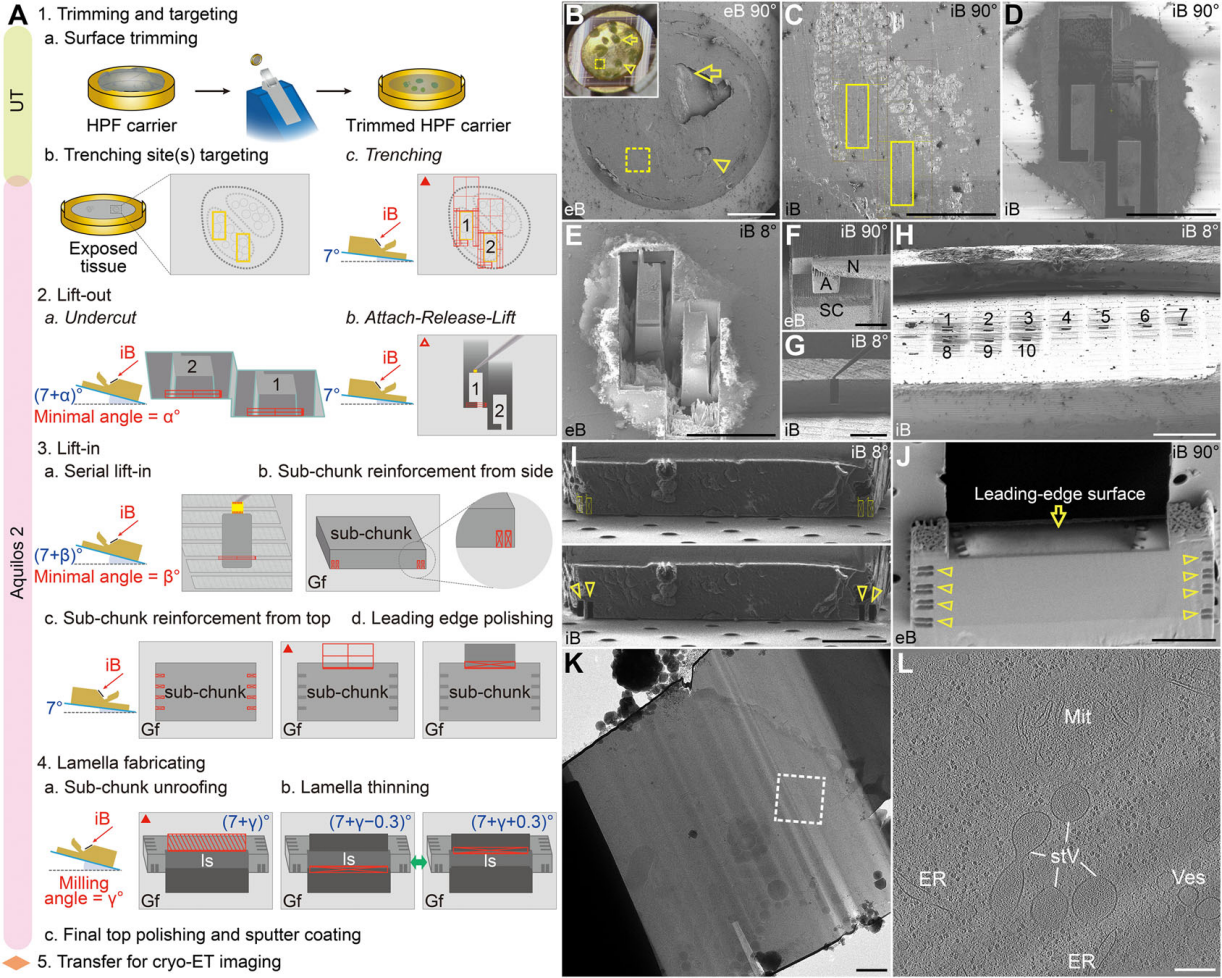
**Application of serialized on‐grid lift‐in sectioning for tomography (SOLIST) to** ***Arabidopsis*** **anthers enables cryo‐ET imaging of developing pollen grains** **(A)** Illustrated workflow of the cryo‐lamellae preparation by SOLIST. Developing anthers are cryo‐fixed by high‐pressure freezing (HPF) in gold‐plated copper carriers (1a left). The carrier is then clamped into the vertical 3‐mm planchette holder of a cryo‐ultramicrotome (UT) for trimming (1a middle), which will make the anthers exposed on the surface of the bulk sample and polish the sample surface (1a right). After transferring the sample into Aquilos 2 chamber, fine target the anther of interest by comparing the sample surface under stereomicroscope and the SEM (1b, interested lift‐out areas containing multiple pollen grains were marked by yellow boxes**)**. Material surrounding the interested lift‐out areas will be removed in the trenching step (1c, milling patterns are indicated by red boxes**)**, when the stage remains at the position enabling the ion beam perpendicular to the sample surface (iB 90° position). Optionally, the sample surface can be coated with a protective organometallic platinum (Pt) layer before trenching. Rotate the stage 180° relative to the iB 90° position and increase the stage tilt by a minimal angle (α°) to let the ion beam mill through the isolated lift‐out area(s) (2a). Briefly sputter‐coat the sample to reduce the charging effects and go to the iB 90° position to attach the needle‐adaptor to the isolated sample chunk by redeposition milling, followed by a release cut at the connecting handle between the lift‐out area and the bulk of the sample to make the sample chunk retractable (2b). Rotate the stage 180° relative to the iB 90° position and increase the stage tilt by a minimal angle (β°) to make the lift‐in landing position visible in the field of view and release the sample sub‐chunks one by one (3a), during which the sub‐chunks can be reinforced by redeposition milling against the grid film (Gf) (3b). After the whole sample chunk has been released on the grid, go to the iB 90° position to reinforce the sub‐chunks from top (3c). The sub‐chunks will be coated with a protective Pt layer before making an opening window and a polished leading‐edge surface (ls) for each sub‐chunk, during which the newly exposed sample surfaces can serve as a reference for sub‐chunk screening (3d). Rotate the stage 180° relative to the iB 90° position and coat a protective Pt layer to protect the leading‐edge surface. Increase the stage tilt by a minimal milling angle (γ°) to unroof the top of the sub‐chunks, during which the newly exposed sample surfaces can serve as a reference for sub‐chunk screening (4a). Remaining sub‐chunks will be subjected to lamellae thinning (4b) and final polishing (4c). After all the sub‐chunks are processed to final lamellae, a short sputter coating will be conducted (4c) to increase the conductivity of the lamellae for further cryo‐TEM imaging (5). **(B)** Location of an anther in the pretrimmed HPF carrier under the SEM. Inlet shows the same carrier under the stereomicroscope above the UT's cryo‐chamber. Arrows and arrowheads indicate two different anthers. **(C)** Ion beam imaging of the dashed box in **(B)**. Target lift‐out areas are indicated by yellow boxes. **(D)** Ion beam imaging showing the two isolated lift‐out areas after the trenching step. **(E)** SEM imaging showing one of the lift‐out areas has been slit by an undercut. **(F)** SEM imaging showing the needle‐adaptor approaching the sample chunk (SC). A, adaptor; N, needle. **(G)** Ion beam imaging showing the lift‐out sample chunk approaching the targeted lift‐in landing grid. **(H)** Ion beam imaging showing a series of sample sub‐chunks on the grid. Numbers indicate the order of sub‐chunk landing. **(I)** Ion beam imaging before (top) and after (bottom) reinforcement redeposition milling at the side of a sub‐chunk. Arrowheads indicate the reinforcement redeposition milling sites. **(J)** SEM imaging showing the leading‐edge surface of a sub‐chunk after leading‐edge polishing. Arrowheads indicate the reinforcement redeposition milling sites. **(K)** Cryo‐TEM overview of a lamella prepared by SOLIST. **(L)** Tomographic slice of the cryo‐electron tomogram generated from the boxed area in **(K)**. Red triangles in **(A)** indicate the protective platinum coating step before milling. The open triangle in **(A)** indicates the important conductive sputter coating step before the attachment of needle‐adaptor to the sample chunk. For **(B**–**J)**, the imaging beam source in the Aquilos 2 chamber is indicated at the lower left corners, and the angle between a beam source and the sample/grid surface is indicated at the upper right corners. eB, electron beam; ER, endoplasmic reticulum; iB, ion beam; Mit, mitochondrion; stV, putative storage vacuoles; Ves, vesicles. Scale bars, 500 μm in **(B)**; 100 μm in **(C**–**E**, **G)**; 10 μm in **(F)**; 200 μm in **(H)**; 5 μm in **(I**, **J)**; 1 μm in **(K)**; 200 nm in **(L)**.

HPF shuttle with a 45° pre‐tilt was used in this workflow. Unlike the Autogrid shuttle, the HPF shuttle contains two different pockets—one for the HPF carrier and the other for the Autogrid. The high‐pressure‐frozen sample underwent the following steps for lamellae preparation: (1) Trimming and targeting, (2) Lift‐out, (3) Lift‐in, and (4) Lamella fabricating. An empty grid clipped into Autogrid was loaded together with the trimmed HPF carrier. Several efforts were made to facilitate successful sample trimming by cryo‐ultramicrotomy (Figure 6A1a) and to maintain the cryo‐fixed bulk sample at well‐vitrified state. First, the HPF carrier was made of copper instead of aluminum to protect the diamond knife for trimming. Second, the bottom of the hat HPF carrier in the HPF assembly was polished by sandpapers and metal polish and treated with 1‐hexadecene or soy lecithin before HPF for better disassembly of the hat carrier and the sample‐loading carrier (Kelley et al., 2022; Nguyen et al., 2024). Third, a thicker cryo‐protectant (e.g., 20% BSA) was used to reduce the brittleness of the cryo‐bulk for better trimming, which prevented the anthers from spalling off during trimming. This also facilitated better vitrification. The aims of the trimming step are to locate/expose the sample on the trimmed surface of the cryo‐bulk and to generate a relatively flat and smooth surface for later manipulation under the dual‐beam microscope. An overview of the trimmed surface under the stereomicroscope of the cryo‐ultramicrotomy (inlet of Figure 6B) helped in the sample targeting under the SEM (Figure 6A1b, B, C). Locules of a trimmed anther can be distinguished under SEM or iB view (Figure 6C), with which we could locate our target lift‐out area(s) containing pollen grains (yellow boxes in Figure 6C). Under the geometry of iB perpendicular to the sample surface (iB 90°), the surrounding material of the target lift‐out areas was milled away with groups of *Regular Cross Section Pattern*s in the trenching step, leaving a handle for each target lift‐out area (Figure 6A1c, D). Importantly, the sample surface should be coated with a protective Pt coating by GIS before trenching. This helps to protect the sample from damage caused by high‐current ion beams. If multiple target lift‐out areas are present, their profiles should be collected prior to GIS coating, as the coating significantly obscures the sharp topology of the trimmed sample surface. Prominent reference markers that remain unaffected by GIS coating can be created via iB milling adjacent to the target lift‐out areas to aid locating after sample reloading.

The stage was then changed to an undercut position (minimal angle without obscuring the milling area) to separate the sample chunk to be lifted out and the deeper bulk sample by FIB‐milling (Figure 6A2a, E). Afterwards, the stage was back to iB 90° position for needle‐adaptor attachment to the sample chunk and a release‐cut at the handle of the sample chunk, followed by lift‐out of the sample chunk by needle manipulation (Figure 6A2b, F). All attachments and welding were operated by GIS‐free redeposition method, forming comb‐like patterns at the position of attachment (Klumpe et al., 2022). The adaptor was pre‐made using a workflow similar to the loop of trench‐undercut‐attach‐release‐lift applied to the cryo‐bulk sample, except that the object was empty grid bars and the attachment positions differed. This step was conducted under cryo‐condition (making the adaptor under RT tended to cause adapter loss after changing to cryo‐condition). Importantly, the sample was sputter‐coated with a conductive Pt coating right before the needle‐adaptor attachment to the sample to reduce the influence of charging on the lift‐out stability.

After locating the target row of the lift‐in grid to its eucentric height, the sample chunk was released in a serial manner at a minimal stage tilt (Figure 6A3a, G, H). Under the same geometry (lift‐in stage position), each released sub‐chunk was reinforced from the front side of the sub‐chunk by GIS‐free redeposition (Figure 6A3b, I). The stage was then changed to iB 90° position for reinforcement from the top of the sub‐chunks, opening, and leading‐edge polishing of the sub‐chunks (Figure 6A3, J). Importantly, before the opening of the sub‐chunks, the sub‐chunks were manually GIS‐coated with a protective Pt layer at the default deposition position to avoid milling curtaining on the leading‐edge surface (Figure 6J). Cellular features could be exposed on the newly generated leading‐edge surface, which also helped in screening of the released sub‐chunks. After manually coating the sub‐chunks at the lift‐in stage position (mind the distance between the shuttle/grid and the GIS needle tip) to protect the newly exposed leading‐edge surfaces, the sub‐chunks were unroofed by *Rectangle Pattern*, which also helped in screening (Figure 6A4a). The remaining sub‐chunks were then subjected to regular lamella thinning and polishing and were sputter‐coated for subsequent cryo‐ET imaging (Figure 6A4, K). Near‐native subcellular structures such as transport vesicles, storage vacuoles with intraluminal vesicles, rough endoplasmic reticulum, and mitochondrion can be identified in the reconstructed cryo‐tomogram (Figure 6L).

### Cryo‐FIB‐SEM volume imaging of plunge‐frozen pollen tubes and high‐pressure‐frozen pollen grains in anthers

Although cryo‐ET excels at resolving *in situ* structures with unprecedented resolution, most of the sample material is lost due to the limited final thickness of the lamellae. The imaging volume is thus restricted to the thin (100–200 nm) lamellae. Additionally, lamellae wider than 10 μm with asymmetric shapes are prone to bending. In this regard, cryo‐FIB‐SEM remains a powerful tool for imaging large, continuous volumes *in situ*. Although it suffers from charging effects and relatively low resolution, it is useful for quick sample screening. Cryo‐FIB‐SEM is one of the imaging modalities of Aquilos 2 Cryo‐FIB (Figure 2), and we also tested its applications to plunge‐frozen pollen tubes and pollen grains in high‐pressure‐frozen anthers.

**Figure 7:**
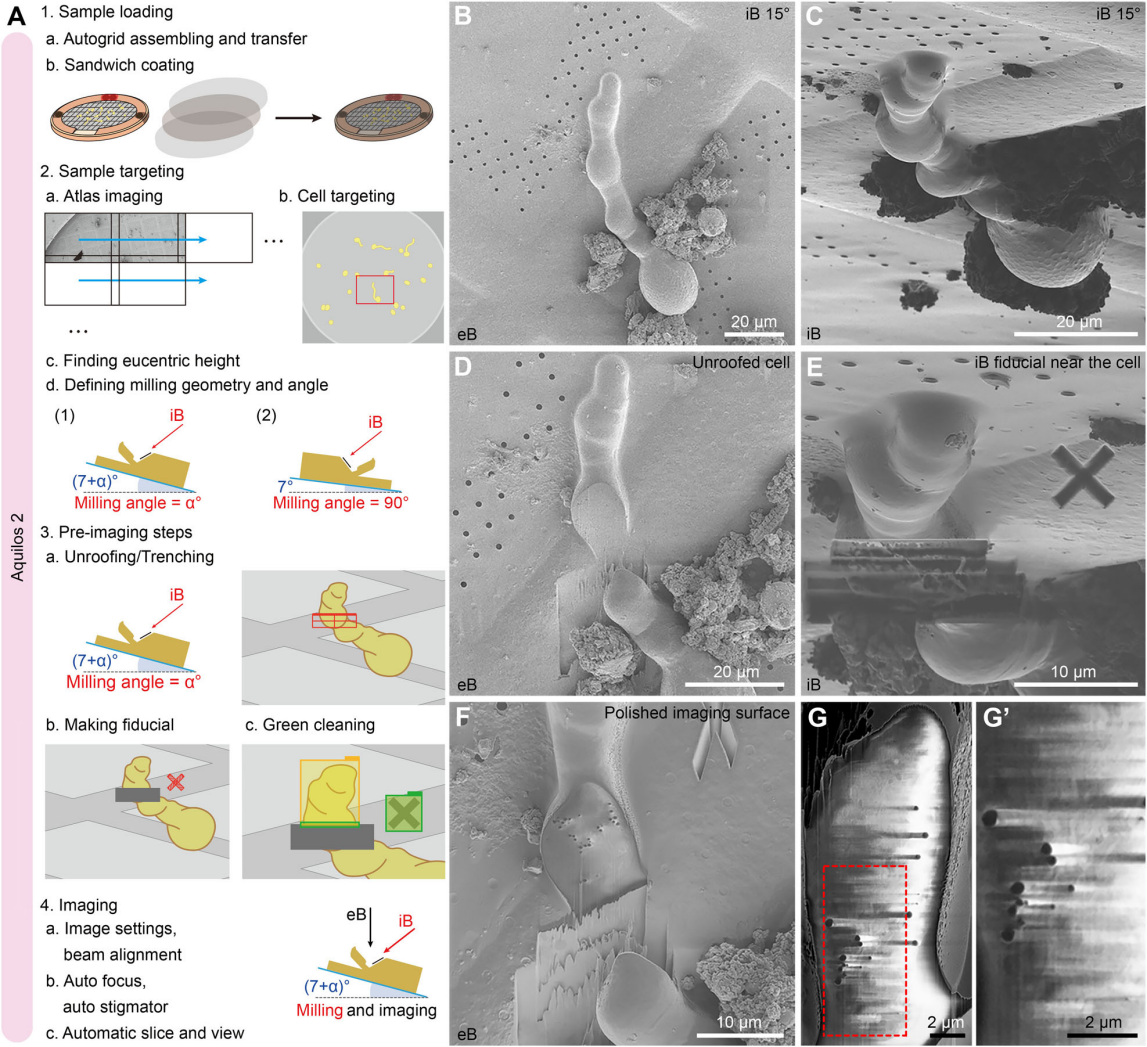
Cryo‐FIB‐SEM volume imaging of plunge‐frozen pollen tubes on a grid **(A)** Illustrated workflow of the cryo‐FIB‐SEM volume imaging of plunge‐frozen pollen tubes. Assembled Autogrid bearing *in vitro*‐cultured *Arabidopsis* pollen tubes is loaded into the Aquilos 2 chamber and subjected to sandwich coating (1). Interested cells will be targeted during Atlas imaging using the MAPS software at 0° scan rotation (2a, b). Define the eucentric height and milling angle for the interested cells according to the geometry of the cells (2c, d). Different from RT‐FIB‐SEM, cryo‐FIB‐SEM usually keeps the stage at a fixed position once the milling/imaging geometry is set. Usually, shallow milling geometry provides a larger imaging window for better electron beam imaging (the first option in 2d) compared to the perpendicular milling geometry (the second option in 2d). The target cell can be unroofed to form an imaging surface (3a). A fiducial marker will be created near the milling area (3b), followed by imaging surface polishing (*Green Clean*) (3c). After proper image settings and beam alignment, the automated FIB‐milling and image collection can be started at the fixed stage tilt angle (4). eB, electron beam; iB, ion beam. **(B**, **C)** A targeted pollen tube with sandwich coating before milling under electron beam **(B)** and ion beam **(C)**. The angle between the ion beam and the grid is indicated at the upper right corners. **(D)** An imaging window or surface is created by ion beam milling. **(E)** Fiducial marker for ion beam milling near the milling area. **(F)** Polished imaging surface after the green cleaning. **(G)** A representative SEM image of the serial raw output images. **(G’)** Enlarged view of the boxed area in **(G)**. Scale bars, 20 μm in **(B**–**D)**; 10 μm in **(E**, **F)**; 2 μm in **(G**, **G’)**.

For plunge‐frozen *Arabidopsis* pollen tubes, sample loading and targeting steps were similar to those of the cryo‐lamellae preparation workflow (compare Figures 5, 7). However, the GIS coating in the cryo‐FIB‐SEM workflow should be thicker than that in the regular cryo‐lamellae preparation because the sample will be exposed to hundreds to thousands of cycles of iB illumination (Figure S3). To maintain optimal scanning‐imaging quality under cryo‐condition, the stage was kept at a fixed position during the loop of milling and imaging, which was different from the restless stage in RT‐FIB‐SEM. Two different stage geometries can be opted for cryo‐FIB‐SEM imaging (Figure 7A2d). One is at a shallow milling angle (left of Figure 7A2d), the other is at a perpendicular milling angle (right of Figure 7A2d). The former one has a more open imaging window, which is better for secondary electron detection. We illustrated the shallow milling angle for this workflow. Pollen tubes displayed undulating morphology on the grid, with bulges and narrower regions (Figure 7B, C). This is a common morphology of the *Arabidopsis* pollen tubes in our plunge‐frozen samples (Figure 5C), which is also evident in our recent study using plunge‐frozen *Arabidopsis* pollen tubes (Figure S14 in Liu et al., 2025). Light microscopy revealed that pollen tubes often grow with an undulating morphology characterized by alternating bulges and constricted regions along their length (Vogler et al., 2015). Certain mutants exhibited bulging morphologies more frequently (H. Li et al., 2018). For small pollen tubes, morphology can be readily assessed on the grid following PF. A target cell was selected, and the milling angle was set as 15° (stage tilt was fixed at 22°) (Figure 7B, C). The cell was first unroofed by FIB‐milling to create a rough imaging surface (Figure 7A3a, D). A fiducial marker was created next to the expected milling area (Figure 7A3b, E). It was preferred to set the marker on beam resistant area (i.e., grid bar) rather than the grid film to avoid the identification error in ASV caused by film distortion. The imaging surface was then polished by the *Green Clean* function of ASV (Figure 7A3c, F). After selection of detector and *Use Case* mode and fine‐tuning the beam geometry in xT UI, key settings such as FIB‐milling parameters, ROI, and SEM scan parameters were defined in the corresponding tabs of the ASV software for automation (Figure 7A4). Figure 7G shows a raw SEM image of the serial dataset, with an enlarged view (Figure 7G’) showing the severe charging effects near the lipid droplets.

**Figure 8:**
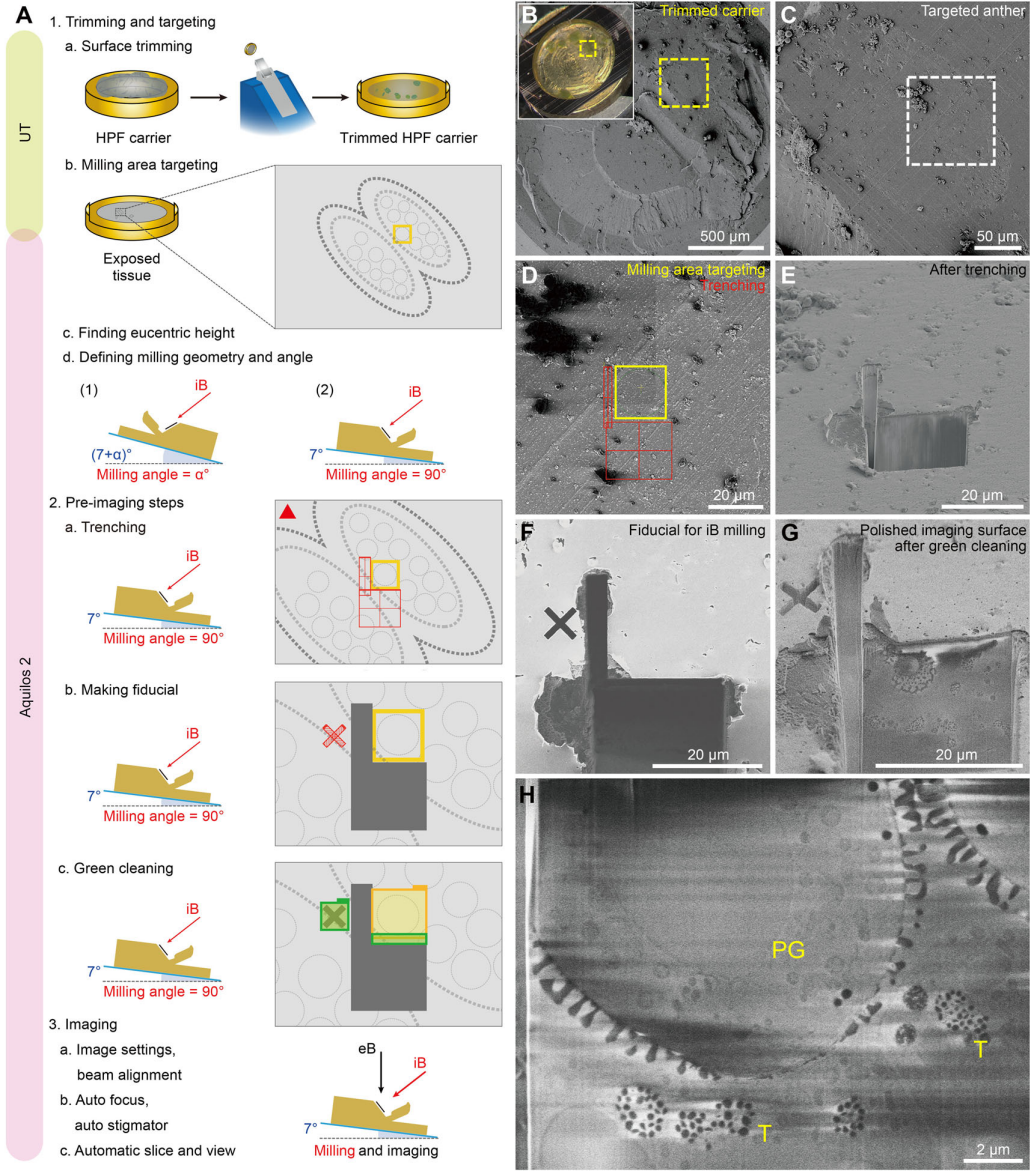
Cryo‐FIB‐SEM volume imaging of pollen grains from high‐pressure‐frozen anthers **(A)** Illustrated workflow of the cryo‐FIB‐SEM volume imaging of developing pollen within anthers. Cryo‐fixed anthers prepared by high‐pressure freezing (HPF) in gold‐plated copper carriers are trimmed by a cryo‐ultramicrotome (UT) to make the anthers exposed on the surface of the bulk sample and to polish the sample surface (1a). After transferring the sample into the Aquilos 2 chamber, routinely coat the sample surface with conductive sputter coating, and target the anther of interest by comparing the sample surface under stereomicroscope and SEM (1b), the imaging area with a pollen grain near the anther wall was marked in the yellow box. Define the eucentric height and milling angle for the interested pollen (1c, d). Stage position in cryo‐FIB‐SEM remains at a fixed position once the milling/imaging geometry is set. And the sample surface should be coated with a protective layer of organometallic platinum (highlighted with the red triangle) and a conductive sputter coating before the next steps. Take the second option in (1d) for example, the stage remains at the position enabling the ion beam perpendicular to the sample surface (iB 90° position), and material surrounding the interested imaging area will be removed in the trenching step (2a, milling patterns are indicated by red boxes). A fiducial marker will be created near the milling area (2b), followed by imaging surface polishing (*Green Clean*) (2c). After proper image settings and beam alignment, the automated FIB‐milling and image collection can be started at the fixed stage position (3). eB, electron beam; iB, ion beam. **(B)** Location of an anther in the pretrimmed HPF carrier under the SEM. Inlet shows the same carrier under the stereomicroscope above the UT's cryo‐chamber. **(C)** Ion beam imaging of the dashed box in **(B)**. **(D)** Enlarged view of the dashed box area in **(C)**. Yellow box indicates the targeted pollen for milling and imaging. Red boxes indicate the milling patterns. **(E)** SEM imaging showing the target area after the trenching step. **(F)** Fiducial marker for ion beam milling near the milling area under ion beam imaging. **(G)** SEM imaging showing the polished imaging surface after the green cleaning. **(H)** A representative SEM image of the serial raw output images. PG, pollen grain; T, tapetum. Scale bars, 500 μm in **(B)**; 50 μm in **(C)**; 20 μm in **(D**–**G)**; 2 μm in **(H)**.

For high‐pressure‐frozen anthers, sample trimming and targeting steps are similar to those of the lift‐out‐aided cryo‐lamellae preparation workflow (compare Figures 6, 8). An exposed anther was located under the SEM with the help of the overview of the trimmed carrier obtained using the stereomicroscope during cryo‐ultramicrotomy (Figure 8A1, B, C). The second stage geometry (iB perpendicular to the sample surface) was illustrated for this workflow (right of Figure 8A1d). One expected milling/imaging area containing a pollen grain was targeted (yellow box in Figure 8D). Material surrounding the target milling/imaging area was then milled away with a group of *Regular Cross Section Pattern*s in the trenching step, creating a vertical imaging surface (Figure 8A2a, D, E). Importantly, the sample surface should be sufficiently GIS‐coated before trenching to protect the sample from high current iB damage during trenching and repetitive iB milling afterwards. A fiducial marker was set next to the expected milling area (Figure 8A2b, F). The imaging surface was then polished by the *Green Clean* function of ASV (Figure 8A2c, G). After selection of the detector and *Use Case* mode and fine‐tuning the beam geometry in xT UI, key settings were defined in the ASV software for automation (Figure 8A3). Another important issue in FIB‐SEM imaging using non‐perpendicular beam geometry (where eB is not normal to the imaging surface) is that the interested area will gradually slide out of the SEM scanning area due to the trigonometric relationship between eB and the imaging surface, as well as the limited beam shift of eB. Proper settings of the *Tilt Correction* (in xT UI) and the *Sample Pre‐tilt* (in ASV) will alleviate the Y‐shift of the image (Figure S4). Figure 8H is a raw SEM image of the serial dataset, showing the target pollen grain (PG) surrounded by tapetum (T) cells.

## DISCUSSION

In this study, we summarized the logic of different imaging modalities in EM‐based 3D subcellular imaging and used *in vitro*‐cultured pollen tubes and developing anthers to demonstrate different sample preparation workflows and use cases based on Aquilos 2 Cryo‐FIB, the commercial dual‐beam microscope with basic integrated functional designs. Our study provides a timely application frame of this accessible system (rather than “home‐made” or other prototype microscopes), beneficial to the plant biologist community.

The acquisition and maintenance of an Aquilos 2 system is comparable to the cost of several large‑scale sequencing projects or a major national research grant, yet its adoption has rapidly expanded worldwide. Leading facilities in Europe, North America, and Asia—including, but not limited to, EMBL, University of Basel's BioEM Lab, HHMI Janelia, Cryo‑Electron Microscopy Center (cEMc) at Stanford University, Center for Biological Imaging (CBI) at the CAS Institute of Biophysics, and the Center for PanorOmic Sciences (CPOS) at The University of Hong Kong, as well as top Chinese mainland universities such as Peking University, Tsinghua University, Sun Yat‑sen University, and Southern University of Science and Technology—have integrated Aquilos 2 into their cryo‑EM platforms. Most facilities maintain a small team of trained staff who act as gatekeepers and trainers, with external users relying on them until the users themselves gain sufficient competence. Basic operations, such as sample loading, cryo‑stage handling, and further lamella fabrication or FIB‐SEM imaging, can typically be mastered within several weeks to two months, depending on prior experience, whereas full utilization of advanced functions (e.g., cryo‐lift‐out) requires 6–12 months of consistent practice. Importantly, these installations highlight the growing emphasis on regional collaboration, with universities and research centers coordinating access, training, and funding to enhance both accessibility and impact across the broader scientific community, while avoiding unnecessary duplication of costs.

From the applications illustrated in this study, the RT‐imaging methods and the cryo‐imaging methods complemented each other in many ways. RT‐imaging methods can obtain larger imaging volumes, and the plastic‐ultrathin‐section‐based RT‐imaging methods allow for multiple rounds of imaging under different magnifications, the so‐called hierarchical imaging (Micheva et al., 2024; Liu et al., 2025). While cryo‐imaging methods generate near‐native ultrastructure with higher accuracy for the interpretation of specific structure and the potential sub‐nanometer resolution (Ader et al., 2019; Klumpe et al., 2025). On the other hand, it is important to choose a proper imaging method and modality according to the sample size, the target ROI, specific subcellular organelles, and the final expected resolution to balance the trade‐off between imaging volume and resolution (Peddie et al., 2022; McCafferty et al., 2024; Zhao et al., 2024). For example, FIB‐SEM and ET imaging of the tip‐vesicles in growing pollen tubes serve different purposes. FIB‐SEM is preferred for visualizing the global distribution of a particular vesicle population because it has a relatively large ROI compared to ET (Weng et al., 2025). While ET is advantageous for classifying distinct vesicle types. (Liu et al., 2025).

Another important step in all these 3D subcellular imaging methods is sample localization and orientation. For example, if one is specifically interested in the cortex cell layer of the root tip, the cell layer should be distinguished under the chosen imaging modality. For some methods in specific cases in our study, such as the RT‐FIB‐SEM imaging of the resin‐embedded anthers and the lift‐out‐aided cryo‐lamellae preparation for cryo‐ET imaging, the samples should be aligned/trimmed in a specific direction and located properly. While for other cases, such as imaging of the pollen tube tips when the perfect apex region is hard to image by cryo‐ET due to the thick supporting material adjacent to the thin lamella area, the solution can be simply increasing the sample quantity to encounter pollen tubes in a better direction relative to iB. These practical issues will also be encountered by other users when these workflows are expanded to other cell types or tissues/organs based on specific biological questions. Therefore, customization is required for different samples. Techniques in different imaging modalities based on the same principle can be mutually applicable. And other elegant workflows, such as the waffle method and serial lift‐out, can be utilized or hybridized with the illustrated workflows using other samples (Kelley et al., 2022; Schiøtz et al., 2024; Pöge et al., 2025; Tang et al., 2025).

**Figure 9:**
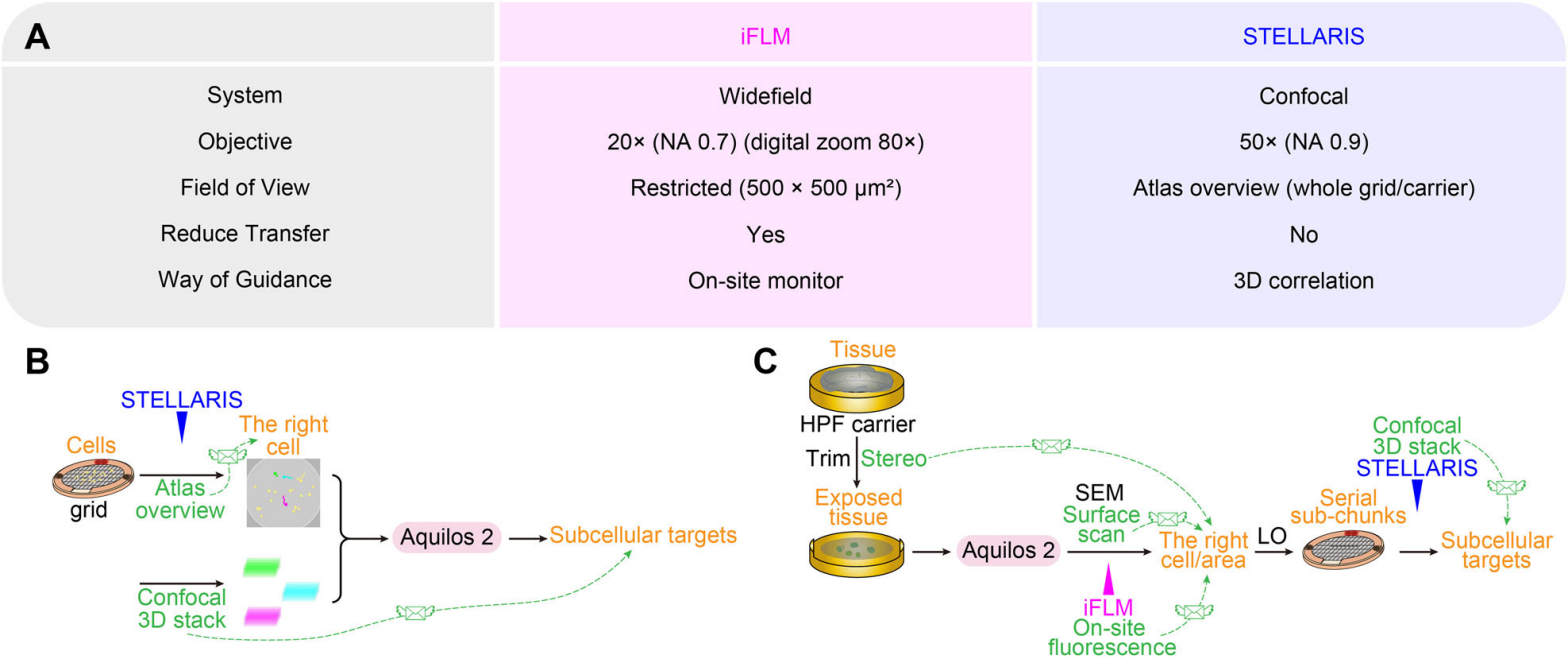
Comparison of the iFLM system for Aquilos 2 and the STELLARIS cryo confocal microscope in the application of cryo‐CLEM to plant materials **(A)** Comparison of the Thermo Fisher Scientific integrated fluorescence light microscope (iFLM) for Aquilos 2 Cryo‐FIB and the Leica STELLARIS cryo confocal microscope. The iFLM system is an upgrade package for the Aquilos 2 Cryo‐FIB. The integrated design aims to reduce the risk of sample contamination during sample transfer. It also aids in monitoring the fluorescence signal of the remaining samples during the lift‐out and lamella fabrication. However, the restricted stage flexibility in the Cryo‐FIB column hinders the fluorescence examination of the whole grid/carrier. Recent upgraded version has improved function in tiled Atlas imaging of the whole grid/carrier. Other stage geometries allowing fluorescence view at the angle of iB view of the milling area require future upgrades of the iFLM or additional script(s) in communication with the microscope. While the confocal system cannot eliminate the risk of ice contamination during sample transfer, it can routinely screen the whole grid in the atlas mode and record the 3D confocal stacks to guide subsequent 3D correlation. **(B**, **C)** Proposed application of the two complementary cryo‐CLEM systems to plunge‐frozen plant cells on grid **(B)** and high‐pressure‐frozen plant tissues **(C)**. Sample types and states are indicated in orange. The two microscope systems and their application nodes for cryo‐CLEM are highlighted with thin magenta and blue arrowheads, respectively. Green words and lines indicate useful information for the correlation guidance. LO, lift‐out; SEM, scanning electron microscopy; stereo, stereomicroscope.

In terms of sample localization, besides the macroscopic/mesoscopic features obtained by EM that help, CLEM is the solution based on molecular markers. Various CLEM strategies have been reported for RT‐ and cryo‐ conditions (Arnold et al., 2016; Bieber et al., 2021; Daraspe et al., 2025; Fung et al., 2023; Heiligenstein et al., 2021; Hoffman et al., 2020; Klein et al., 2021; Kukulski et al., 2011; S. Li et al., 2023a; W. Li et al., 2023; Wang et al., 2024). These can be classified from different aspects: RT‐/cryo‐ correlation, pre‐/post‐fixation correlation, 2D/3D correlation. Most importantly, we should focus on the dimension scale and resolution of the correlation and ask whether they are suitable for our interested samples. When interested in subcellular organelles, two popular commercial post‐fixation correlation systems for cryo‐CLEM were compared in Figure 9A. Based on our preliminary tests and user experience, we proposed the workflows with correlation nodes suitable for the plant materials used in this study (Figure 9B, C). There are indeed other systems, such as Meteor 2.0 (https://www.delmic.com/en/products/cryo-solutions/meteor), cryo‐STAR (S. Li et al., 2023b), HOPE‐SIM (S. Li et al., 2023a), and cryo‐CLIEM (W. Li et al., 2023), which still need to be popularized and validated using diverse types of samples. Whether a system can truly withstand the demands of routine application depends on testing outcomes with real biological specimens and collaborative projects with joint effort. We believe the popularization of these technologies in the plant biology community will lead us to a more comprehensive and native understanding of plant cells.

## MATERIALS AND METHODS

### Plant materials and pollen germination

*Nicotiana tabacum* plants were cultured in a greenhouse (22°C; 12 h/12 h of light/darkness). *Arabidopsis thaliana* (Col‐0) plants were grown in a plant growth room (22°C; 16 h/8 h of light/darkness). Pollen grains were freshly collected from opening flowers before germination in *in vitro* germination media (GM) (tobacco GM: 0.01% (w/v) H_3_BO_3_, 1 mM CaCl_2_, 1 mM Ca(NO_3_)_2_, 1 mM MgSO_4_, 10% sucrose, pH 6.5; *Arabidopsis* GM: 0.01% (w/v) H_3_BO_3_, 1 mM CaCl_2_, 1 mM Ca(NO_3_)_2_, 1 mM MgSO_4_, 18% sucrose, pH 7) (H. Li et al., 1999; Wang and Jiang, 2011). The tobacco pollen grains were incubated in tobacco GM at 27.5°C for 1.5 h before cryo‐fixation. The *Arabidopsis* pollen grains were incubated in *Arabidopsis* GM at 22.5°C for 6 h before cryo‐fixation. *Arabidopsis* anthers of mixed developing stages were plucked by tweezers or syringe needles right before cryo‐fixation.

### RT‐FIB‐SEM sample preparation and imaging

#### Developing *Arabidopsis* pollen grains

Freshly collected *Arabidopsis* anthers of mixed developing stages were subjected to HPF (Leica EM ICE) with 0.15 M sucrose as cryo‐protectant in type B aluminum HPF carrier (Leica #16770142), followed by FS (Leica EM AFS2) in acetone containing 2% OsO_4_. FS was conducted according to a previous study (Czymmek et al., 2020). Durcupan™ ACM (EMS #14040) was used for resin infiltration and embedding according to the product instructions. A cured resin block was glued on the 45° slope of low profile 45°/90° SEM pin stub (Ted Pella #16104) by Aron Alpha® Instant Glue and trimmed by ultramicrotomy (Leica UC7). Thin sections peeled off from the top and side surfaces of the block were examined under 80 kV 2D TEM (Hitachi H‐7650). Base area of the trimmed block without sample was painted by Fast Drying Silver Paint (Ted Pella #16040‐30). And the pin stub with sample was subjected to sputter coating (Edwards S150B, gold/palladium source) for 1 min. The sample was transferred into the Aquilos 2 chamber with a standard shuttle.

The temperature of the GIS source was 45°C. *Scan Rotation* was off (0°) throughout the following steps. The trimmed block edge was aligned to the horizontal by the *Align Feature* function, and the edge with the interested sample area was located to its eucentric height. The top and side surfaces of the interested sample area were further trimmed by FIB‐milling (*Cleaning Cross Section Pattern* [*CCS*], 30 kV, 30 nA) to create orthogonal surfaces strictly perpendicular to the corresponding beams. At the stage position where iB is parallel with the SEM imaging area, milling area (34 × 20 μm^2^) and fiducial area (15 × 15 μm^2^) for FIB‐milling were coated by beam‐induced GIS‐coating (*Rectangle Pattern*, *Application*: *Pt dep*, 16 kV; 3.6 nA and 2.5 nA respectively). *Z Size* of the patterns was 5 μm for the coating of the milling area, and 2 μm for that of the fiducial area. Material surrounding the milling area was cleaned by FIB‐milling (*CCS*, 30 kV; 65 nA for rough milling, 30 nA for fine milling). SEM imaging surface was polished by FIB‐milling (*CCS*, 30 kV, 5 nA). At the stage position where iB is perpendicular to the SEM imaging surface, a fiducial area (12 × 12 μm^2^) for SEM imaging was coated by beam‐induced GIS‐coating (*Rectangle Pattern*, *Application*: *Pt dep*, 16 kV, 2.5 nA, *Z Size* 2 μm). At the corresponding stage positions, mill the fiducial markers for FIB‐milling and SEM imaging with distinct patterns (30 kV, 1 nA, *Application*: *Si*, *Z Size* 3 μm [> *Z Size* of the GIS‐coating for fiducial areas]).

A new project was created in ASV 4.2.4 (TFS). At the stage position where iB is parallel with the SEM imaging surface, the fiducial for FIB‐milling and the FIB‐milling area were defined in the *MILLING* tab. The imaging surface was subjected to *Green Clean* in the *SAMPLE PREPARATION* tab with temporary *Slice Thickness* (in the *MILLING* tab) of 50 nm and the *ROCKING MILL* (in the *MILLING* tab) on (*Tilt Angle* 5°). The *Slice Thickness* was then redefined as the expected milling step (5 nm), and the *Depth* of milling was set to 80 μm. The ion beam for milling was at 30 kV, 1 nA. In xT UI 20.1.1 (TFS), at the stage position where eB is perpendicular to the SEM imaging surface, *Mode 2: OptiPlan* was selected in *Use Case,* and *T1* was selected as the detector (*Detector Settings*: *A* + *B*) for imaging. Stage Z was re‐linked to FWD (Free Working Distance) after re‐adjustment of the focus near the target imaging area. The Z position of the stage was decreased to around 3 mm (sample block should not touch the pole piece) with the *Z‐Y Link* ticked. Image condition was optimized by fine‐tuning the beam geometry in *Direct Adjustments*, focus, and the stigmator. The *Inverse* option was ticked in the *LUT* (*Look‐Up‐Table*) tab.

*SEM Image* was set in the *IMAGING* tab of the ASV. SEM fiducial and scan resolution were defined (*SEM Image* setup: 4096 × 3536, 16‐bit, 3 μs × 1 (Line integration) × 1 (Frame integration), 20.48 μm in width of *Aligned Image*, equal to 5 nm pixel size in X and Y; *Alignment* setup: 1536 × 1024, 8‐bit, 500 ns × 1 (Frame integration), 2 kV, 0.1 nA). *Auto Focus* and *Auto Stigmator* areas were placed centrally in the vertical dimension of the image area, with overlapping, where the image feature was sharp and clear. The frequency of the *Auto Focus* and *Auto Stigmator* was 5. *Selected Area Scan* was ticked to further decrease the SEM scanning area to save data collection time. After satisfied results of *Auto Focus* and *Auto Stigmator*, the automation project was initiated for the ASV to run.

#### Tobacco pollen tubes

*In vitro*‐cultured pollen tubes were harvested by low‐speed centrifugation. Concentrated pollen tubes in tobacco GM were directly subjected to HPF (Leica EM ICE) using type B HPF aluminum carrier (Leica #16770142). Subsequent FS was performed in acetone containing 2% OsO_4_ (Leica EM AFS2). The FS program was as follows: (1) Stay at −80°C for 54 h (0.1% uranyl acetate was supplied to the FS cocktail 24 h after the program initiation); (2) Gradually warm up to −20°C for 24 h; (3) Gradually warm up to −4°C for 12 h; (4) Gradually warm up to 0°C for no more than 96 h; (5) Transfer samples to 4°C for 10 min. Samples were washed in precooled acetone three times. HPF carriers were removed from samples at the last time of the acetone wash. Samples were infiltrated stepwise in 10%, 25%, 50%, and 75% of Epon812 resin (EMS #14120) (in acetone) for around 48 h in total, infiltrated in 100% Epon812 resin for two times for 24 h in total, and finally cured in 100% Epon812 resin with accelerator at 60°C for 24 h. Cured sample blocks were glued to a flat SEM stub (Ted Pella #16111) and trimmed by ultramicrotomy (Leica UC7). Thin sections were screened by 80 kV 2D TEM (Hitachi H‐7650). Base area without samples was painted with Fast Drying Silver Paint (Ted Pella #16040‐30). After sputter coating (Edwards S150B, gold/palladium source), samples were transferred into the Aquilos 2 chamber with a standard shuttle.

The temperature of the GIS source was 45°C. *Scan Rotation* was off (0°) throughout the following steps. After targeting the milling area to its eucentric height, beam‐induced Pt deposition was conducted on top of the milling area (21 × 19 μm^2^) and fiducial area (12 × 12 μm^2^) for FIB‐milling (*Rectangle Pattern*, *Application*: *Pt dep*, 16 kV; 3.6 nA and 2.5 nA, respectively) at the stage position where iB is perpendicular to the top of the sample block. *Z Size* of the patterns was 5 μm for the coating of the milling area, and 1 μm for that of the fiducial area. Material surrounding the milling area was cleaned by FIB‐milling (*Regular Cross Section Pattern* [*RCS*], 30 kV; 65 nA, *Z Size* 50 μm). The trench was further fine trimmed by FIB‐milling (*CCS*, 30 kV; 65 nA, *Z Size* 50 μm). FIB fiducial was created by FIB‐milling (30 kV, 1 nA, *Z Size* 2 μm). SEM imaging surface was polished by FIB‐milling (*CCS*, 30 kV, 30 nA, *Z Size* 60 μm) until the target cell was exposed on the polished surface. At the stage position where the imaging surface faces the iB, a fiducial area for SEM imaging was coated by GIS (*Rectangle Pattern*, *Application*: *Pt dep*, 16 kV; 2.5 nA, *Z Size* 1 μm). SEM fiducial was created by FIB‐milling (30 kV, 1 nA, *Z Size* 2 μm).

A new project was created in ASV 4.2.4 (TFS). At the stage position where iB is parallel with the SEM imaging surface, the FIB fiducial and the FIB‐milling area were defined. The imaging surface was subjected to *Green Clean* with a temporary *Slice Thickness* of 50 nm. The *Slice Thickness* was then redefined as the expected milling step (5 nm), and the *Depth* of milling was set to 60 μm. The ion beam for milling was at 30 kV, 1 nA. In xT UI, *Mode 3: OptiTilt* was selected in *Use Case,* and *T1* was selected as the detector (*Detector Settings*: *A* + *B*) for imaging. Stage Z was re‐linked to FWD after re‐adjustment of the focus near the target imaging area. The Z position of the stage was decreased to around 3 mm with the *Z‐Y Link* ticked. Imaging condition was optimized by fine‐tuning the beam geometry in *Direct Adjustments*, focus, and the stigmator. The *Inverse* option was ticked in the *LUT* tab. In the *Tilt Correction* tab, *Dynamic Focus* was ticked, and *Automatic (Cross Section)* was selected in *Tilt Angle*. The *SEM Image* was set in the *IMAGING* tab of the ASV. SEM fiducial and scan resolution were defined (*SEM Image* setup: 6144 × 4096, 16‐bit, 3 μs × 1 (Line integration) × 1 (Frame integration), 15.36 μm in width of *Aligned Image*, equal to 2.5 nm pixel size in X and Y; *Alignment* setup: 1536 × 1024, 8‐bit, 500 ns × 1 (Frame integration), 2 kV, 0.1 nA). The *Beam* option was selected in the *Y‐SHIFT CORRECTION*. After satisfied results of *Auto Focus* and *Auto Stigmator*, the automation project was initiated in ASV.

### Cryo‐lamellae preparation and cryo‐ET imaging

#### *Arabidopsis* pollen tubes

Concentrated pollen tubes in *Arabidopsis* GM were directly subjected to PF (TFS, Mark IV Vitrobot). Four microliters of concentrated pollen tubes in *Arabidopsis* GM were supplied on glow‐discharged (PELCO, easiGlow, Ted Pella; 30 mA, 30 s) Quantifoil® SiO_2_ R1/4 Au 200 mesh grid. The sample drop was facing the Teflon side of the blotting arm, with the filter paper side blotting the cell culture from the back of the grid. Grids were then subjected to PF (50%–60% *Humidity*, 22°C, *Blot Time* 10 s, *Blot Force* 10) in liquid ethane.

Aquilos 2 system was purged with cooling N_2_ gas with 0.2 MPa pressure and 190 mg/s flow rate for at least 30 min. The temperature of the GIS source was 25°C. GIS was purged for 30 s, and the sputter coating argon was purged with 0.05 MPa pressure for two cycles. Vacuum mode was changed back to *High Vacuum* after sputter purge. The cryo‐stage and cryo‐shield temperatures were cooled down to around −193°C with the insertion of a heat exchanger into liquid nitrogen. Plunge‐frozen sample grids clipped into Autogrids (TFS, 1205101 and 1036171) were transferred into the Aquilos 2 chamber with a 45° grid shuttle. Grids were sputter‐coated (30 mA, 10 Pa, 1 V) for 12 s. Stage Z was linked to the FWD. Lamella sites and their eucentric heights and milling angles were registered in MAPS 3.14 (TFS). Grids were coated with organo‐platinum by GIS for 30 s to 1 min and were sputter‐coated again (30 mA, 10 Pa, 1 V, 12 s). Both the eB and iB *Scan rotation*s were set to 180°. All the FIB‐milling steps were conducted at 30 kV voltage. Lamellae were milled to around 4 μm at 30 kV, 1 nA with *Rectangle Pattern*. Relief cuts were milled 5 μm off from both left and right ends of each lamella at 1 nA with two *Rectangle Pattern*s. Lamellae were thinned to 400 nm at decreasing current of iB and finally polished close to 100 nm at 10 pA using *CCS* patterns with compensation stage tilt of ± 0.3°. Polished lamellae were sputter‐coated at 7 mA, 10 Pa, 1 V for 10 s.

Autogrids were loaded into a 300 kV Krios G3i cryo‐TEM (TFS) with an energy filter and Gatan K3 direct electron detector. Camera references were taken at the recommended dose rate (15 e^–^/px/s) and applied to the same machine session. Batch tomogram collection was started after the individual eucentric height of each imaging site was found by the *Auto eucentric height* function and registered. Tilt image series were taken by Tomography 5.3 (TFS) at a magnification of 33,000 × at 2.7 Å pixel size using a dose symmetric acquisition scheme with 2° tilting steps and a total dosage below 150 e^–^/Å^2^. The highest angles without obstacles for each tilting series were pre‐examined by manual stage tilt. Tilting range was no more than ± 60°.

#### Developing *Arabidopsis* pollen grains

Freshly collected *Arabidopsis* anthers of mixed developing stages were subjected to HPF (Leica EM ICE) with 20% BSA (in 1× PBS) as cryo‐protectant in type B gold‐plated copper HPF carrier (Leica #16770153). The carrier with the sample was vertically loaded into a cryo‐ultramicrotome (Leica FC7) for top surface trimming (DiATOME trim 45) (feed step 200 nm, speed 0.8 mm/s for rough trim). Optionally, the carrier can be loaded on top of a spacer (Harmony Technology Art.1712) in case of deep sample embedding. Feed and speed of the trimming were decreased to 100 nm and 0.6 mm/s for fine polish when anthers were exposed to the surface of the cryo‐bulk. Polished carrier was imaged under the stereomicroscope of the ultramicrotome and transferred into a prepared Aquilos 2 chamber (GIS temperature 25°C) together with a glow‐discharged (PELCO, easiGlow, Ted Pella; 30 mA, 30 s) Quantifoil® SiO_2_ R1/4 Au 200 mesh grid using a 45° HPF shuttle. Both the eB and iB *Scan rotation*s were set to 180° throughout the following steps.

At the stage position where iB is perpendicular to the empty grid, an adaptor area (8 × 6 μm^2^, this area should roughly match the area of the needle tip) was marked at a grid bar region. Except for the handle (4 × 2 μm^2^) at the left of the adaptor area, material surrounding the adaptor area was removed by FIB‐milling (*RCS*, 30 kV, 5 nA) until the grid bar/film was cut through. Side surfaces of the adaptor were polished by FIB‐milling at a lower current (*CCS*). At the stage position of adaptor undercut (the iB entered through the Autogrid milling slot at a shallow angle), the adaptor was trimmed from the bottom to around 9 μm in height, and the handle was trimmed from the bottom to 2 μm in height. The top of the adaptor was thoroughly polished (*CCS*, 30 kV, 1 nA). The EasyLift needle was inserted and dragged to a position hanging above the adaptor. After polished, the bottom of the needle tip was attached to the top of the adaptor with the front edge of the adaptor top a bit “lower” than the front edge of the needle tip, followed by a welding FIB‐milling on the adaptor near the attaching interface with the needle (GIS‐free redeposition, *CCS*, *Top To Bottom*, 30 kV, 0.1 nA, twice). A second round of redeposition was conducted at the “ridges” left by the first round of redeposition for reinforcement. The handle was then cut off to release the adaptor (30 kV, 1 nA). The released adaptor was lifted out higher, and the bottom of the adaptor was finely polished (*Rectangle Pattern*, 30 kV, 0.5 nA) before the needle was retracted.

After sputter coating (30 mA, 10 Pa, 1 V, 12 s), exposed anthers were documented by eB (2 kV, 13 pA) and iB (30 kV, 10 pA) imaging. An interested anther was further located with the help of the overview under the stereomicroscope. The carrier surface was GIS‐coated at the default *Deposition Position* for 30 s. At the stage position where iB is perpendicular to the carrier surface (iB 90°), target lift‐out areas (each around 25 × 70 μm^2^) were marked, and the surrounding material was removed by FIB‐milling (*RCS*, 30 kV, 3 nA, *Z Size* 3 μm). At the stage position of undercut, the target lift‐out area was separated from the cryo‐bulk by 2–3 times of FIB‐milling (*RCS*, 30 kV, 0.5 nA), with around 9 μm (in height/thickness) sample chunk hanging. At the stage position of iB 90°, the side surface (opposite to the handle) of the hanging chunk was fine polished (*CCS*, 30 kV, 0.3 nA). The carrier was then sputter‐coated to reduce charging effects (30 mA, 10 Pa, 1 V, 12 s). EasyLift needle with polished adaptor was inserted and dragged to touch the polished sample chunk surface with the front edge of the adaptor bottom a bit “lower” than the front edge of the polished chunk surface. After welding FIB‐milling (GIS‐free redeposition, *CCS*, *Bottom To Top*, 30 kV, 0.1 nA, twice; number and size of the *CCS* patterns were scaled up compared to the welding of the needle and the adaptor), the handle of the hanging sample chunk was cut off (*RCS*, *Left to Right* or *Right to Left*, 30 kV, 1 nA, enough *Z size* to make sure the handle was completely broken with only one round of milling) to release the sample chunk. The sample chunk was then lifted out and retracted by the needle.

At the stage position of lift‐in (*Grid 2*, *Milling Angle* 6°), the sample chunk was inserted by the needle. Bottom of the sample chunk was polished to flat (*CCS*, 30 kV, 1 nA) and sub‐chunks (3–5 μm in thickness) were released in a serial manner by FIB‐milling (*RCS*, *Left to Right* or *Right to Left*, 30 kV, 0.5 nA, *Y Size* > 1.5 μm) with each grid square containing one sub‐chunk. In each release, FIB‐milling was conducted when the bottom of the sample chunk was just about to be dragged away from the grid film. During the serial lift‐in, each sub‐chunk was reinforced by GIS‐free redeposition milling at the two corners of the sub‐chunk near the grid film facing the iB (*CCS*, *Bottom To Top*, 30 kV, 10 pA, *Z Size* 0.4 μm, twice; depends on different samples). The needle was retracted after all the sample chunk was released on the grid. The grid was GIS‐coated at the default *Deposition Position* for 20 s. At the stage position of iB 90° (iB perpendicular to the grid surface), sub‐chunks were reinforced by FIB‐milling (*CCS*, *Left to Right* for the left boarder and *Right to Left* for the right boarder, 30 kV, 0.1 nA; depends on different samples). The sub‐chunks were then milled at the stage position of iB 90° to make the milling window (*RCS*, *Top To Bottom*, 30 kV, 0.5–1 nA; avoiding the reinforcement milling areas). The created leading‐edge surfaces were polished by FIB‐milling at a lower current (*CCS*, *Top To Bottom*, 30 kV, 10–50 pA). At the stage position of lift‐in, stage Z was increased to around 11 mm, and stage Y was increased by around 0.4 mm (depending on different machines) with *Z‐Y Link* ticked to make the distance from the grid to the GIS needle tip comparable to that when the stage was at the default *Deposition Position*. The GIS needle was inserted, and the grid was manually GIS‐coated for 20–30 s. At the stage position of milling, cryo‐lamellae preparation was conducted following a procedure similar to that used for the pollen tube samples.

### Cryo‐FIB‐SEM sample preparation and imaging

#### *Arabidopsis* pollen tubes

Cryo‐fixation of the pollen tubes was the same as that in the cryo‐lamellae preparation. Autogrids with samples were transferred into a prepared Aquilos 2 chamber (GIS temperature 25°C) with a 45° grid shuttle. Grids were sputter‐coated (30 mA, 10 Pa, 1 V, 12 s) and GIS‐coated (1 min), followed by a second sputter coating (30 mA, 10 Pa, 1 V, 12 s). *Scan Rotation* was off (0°) throughout the following steps. The target cell at its eucentric height (Stage T was set to a fixed angle throughout the following steps) was opened by FIB‐milling to create a rough imaging surface (*RCS*, *Bottom To Top*, 30 kV, 1 nA), followed by fine polishing of the imaging surface (*CCS*, *Bottom To Top*, 30 kV, 0.3 nA). A fiducial marker was created next to the FIB‐milling area (30 kV, 0.5 nA on grid bars, *Z Size* 2 μm). A new project was created in ASV 4.2.4 (TFS). The FIB fiducial and the FIB‐milling area were defined. The imaging surface was subjected to *Green Clean* with a temporary *Slice Thickness* of 50 nm. The *Slice Thickness* was then redefined as 20 nm and the *Depth* of milling was set to 3 μm (depending on the Y size of ROI). The ion beam for milling was at 30 kV, 50 pA. *Sample Pre‐tilt* was changed to –45° in the *MILLING* tab of ASV. In xT UI, *Mode 1: Standard* was selected in *Use Case,* and *ETD* was selected as the detector for imaging. Image condition was optimized by fine‐tuning the beam geometry in *Direct Adjustments*, focus, and the stigmator. In the *Tilt Correction* tab, *Dynamic Focus* and *Tilt Correction* were ticked, and *Automatic (Cross Section)* was selected in *Tilt Angle* (The angle in the *Manual* should be –38°). SEM scanning pixel width (PW) was tested in xT UI (around 10 nm; scan at 2 kV, 13 pA). *SEM Image* was set in the *IMAGING* tab of the ASV (*SEM Image* setup: 6144 × 4096, 16‐bit, 50 ns × 100 (Line integration) × 1 (Frame integration), *Selected Area Scan* ticked, 10.74 nm pixel size in X and Y). The *Beam* option was selected in the *Y‐SHIFT CORRECTION*. *Auto Focus* area was placed on the interface between the cell area and the GIS‐coating area. The frequency of the *Auto Focus* was 20. After satisfied results of *Auto Focus*, the automation project was initiated in the ASV.

#### Developing *Arabidopsis* pollen grains

Cryo‐fixation of the anthers was the same as that in the cryo‐lamellae preparation. The trimmed carrier was transferred into the prepared Aquilos 2 chamber (GIS temperature 25°C) with a 45° HPF shuttle. *Scan Rotation* was off (0°) throughout the following steps. The sample was sputter‐coated (30 mA, 10 Pa, 1 V, 12 s) and the area with exposed anther was located under SEM with the help of the overview under the stereomicroscope. Carrier was GIS‐coated for 1 min at the default *Deposition Position*. At the stage position of iB 90°, the target milling area was marked, and the surrounding material was removed by FIB‐milling (*RCS*, 30 kV, 3 nA, *Z Size* 5 μm). A fiducial marker was created next to the milling area (30 kV, 0.1 nA, *Z Size* 2 μm). A new project was created in ASV 4.2.4 (TFS). The FIB fiducial and milling area was defined. The imaging surface was polished by *Green Clean* (*Slice Thickness* 50 nm). The *Slice Thickness* was then set to 20 nm, and the *Depth* of milling was set to 5 μm (depending on the Y size of ROI). The ion beam for milling was at 30 kV, 0.1 nA. *Sample Pre‐tilt* was changed to 45° in the *MILLING* tab of ASV. In xT UI, *Mode 1: Standard* was selected in *Use Case,* and *ETD* was selected as the detector for imaging. Image condition was optimized by fine‐tuning the beam geometry in *Direct Adjustments*, focus, and the stigmator. In the *Tilt Correction* tab, *Dynamic Focus* and *Tilt Correction* were ticked, and *Automatic (Cross Section)* was selected in *Tilt Angle* (The angle in the *Manual* should be –38°). SEM scanning pixel width (PW) was tested in xT UI (around 10 nm; scan at 2 kV, 25 pA). *SEM Image* was set in the *IMAGING* tab of the ASV (*SEM Image* setup: 4096 × 3536, 16‐bit, 50 ns × 50 (Line integration) × 1 (Frame integration), *Selected Area Scan* ticked, 10.12 nm pixel size in X and Y). *The beam* option was selected in the *Y‐SHIFT CORRECTION*. *Auto Focus* area was placed on the interface between the cell area and the GIS‐coating area (Frequency 20). After satisfied results of *Auto Focus*, the automation project was initiated in the ASV.

## CONFLICTS OF INTEREST

The authors declare no conflicts of interest.

## AUTHOR CONTRIBUTIONS

Z.Q.L. conducted the sample preparation, FIB‐SEM and cryo‐ET imaging of the pollen tube samples. Z.Z.L. prepared the anther/pollen samples for FIB‐SEM imaging. Z.Q.L. and Z.Z.L performed the SOLIST experiment for cryo‐ET imaging with the help of P.S.E. R.M. contributed to the RT‐FIB‐SEM imaging and regular cryo‐lamellae preparation experiments. Y.X.H. and T.N. conducted the cryo‐ET imaging of the cryo‐lift‐out prepared cryo‐lamellae. M.F.L. and Y.X.H. did the cryo‐ET data analysis. J.Y.G. and W.Q.W. helped with RT sample preparation. Z.Q.L. and L.W.J. wrote and edited the article. All authors have read and approved the contents of this paper.

## Supporting information

Additional Supporting Information may be found online in the supporting information tab for this article: http://onlinelibrary.wiley.com/doi/10.1111/jipb.70143/suppinfo

**Figure S1.** Tomographic slices and the impact of section warping on reconstruction quality
**Figure S2.** Tomographic slices of a joined tomogram covering the tobacco pollen tube tip region
**Figure S3.** Worn protective coating at the interface of the cell and the grid film
**Figure S4.** Compensation for the image Y‐shift by *Sample Pre‐tilt* input

**Movie S1.** Aligned serial scanning electron micrographs of the slices of the *Arabidopsis* pollen grain (Corresponding to Figure 3G)

**Movie S2.** Aligned serial scanning electron micrographs of the slices of the germinating tobacco pollen tube (Corresponding to Figure 4H)

**Movie S3.** Electron tomogram of the lamella prepared from a plunge‐frozen *in vitro*‐cultured *Arabidopsis* pollen tube (Corresponding to Figure 5G)

**Movie S4.** Electron tomogram of the lamella prepared from high‐pressure‐frozen *Arabidopsis* anther (Corresponding to Figure 6L)

**Movie S5.** Aligned serial cryo‐scanning electron micrographs of the slices of the *Arabidopsis* pollen tube (Corresponding to Figure 7G)

**Movie S6.** Aligned serial cryo‐scanning electron micrographs of the slices of the *Arabidopsis* pollen grain (Corresponding to Figure 8H)
**Table S1.** Glossary of terms
