# Supplementary material for: Electron microscopy‐based three‐dimensional subcellular imaging of plant male gametophyte
Source: J Integr Plant Biol. 2026 Jan 21;68(5):1290–314. doi: 10.1111/jipb.70143 (PMC13140077; doi:10.1111/jipb.70143)
Supplement: Supplementary file 1 — Figure S1. Tomographic slices and the impact of section warping on reconstruction quality Figure S2. Tomographic slices of a joined tomogram covering the tobacco pollen tube tip region Figure S3. Worn protective coating at the interface of the cell and the grid film Figure S4. Compensation for the image Y‐shift by Sample Pre‐tilt input [file JIPB-68-1290-s002.docx]

**SUPPORTING INFORMATION**

**Electron Microscopy-Based Three-Dimensional Subcellular Imaging of Plant Male Gametophyte**

Zhiqi Liu^1,2†^*, Zizhen Liang^1,2†^*, Mengfei Liao^1^, Yixin Huang^3^, Rui Ma^4^, Jiayang Gao^1,2^, Weiqi Wang^1^, Tao Ni^3,5^, Philipp S. Erdmann^6^*, and Liwen Jiang^1,2,7,8^*


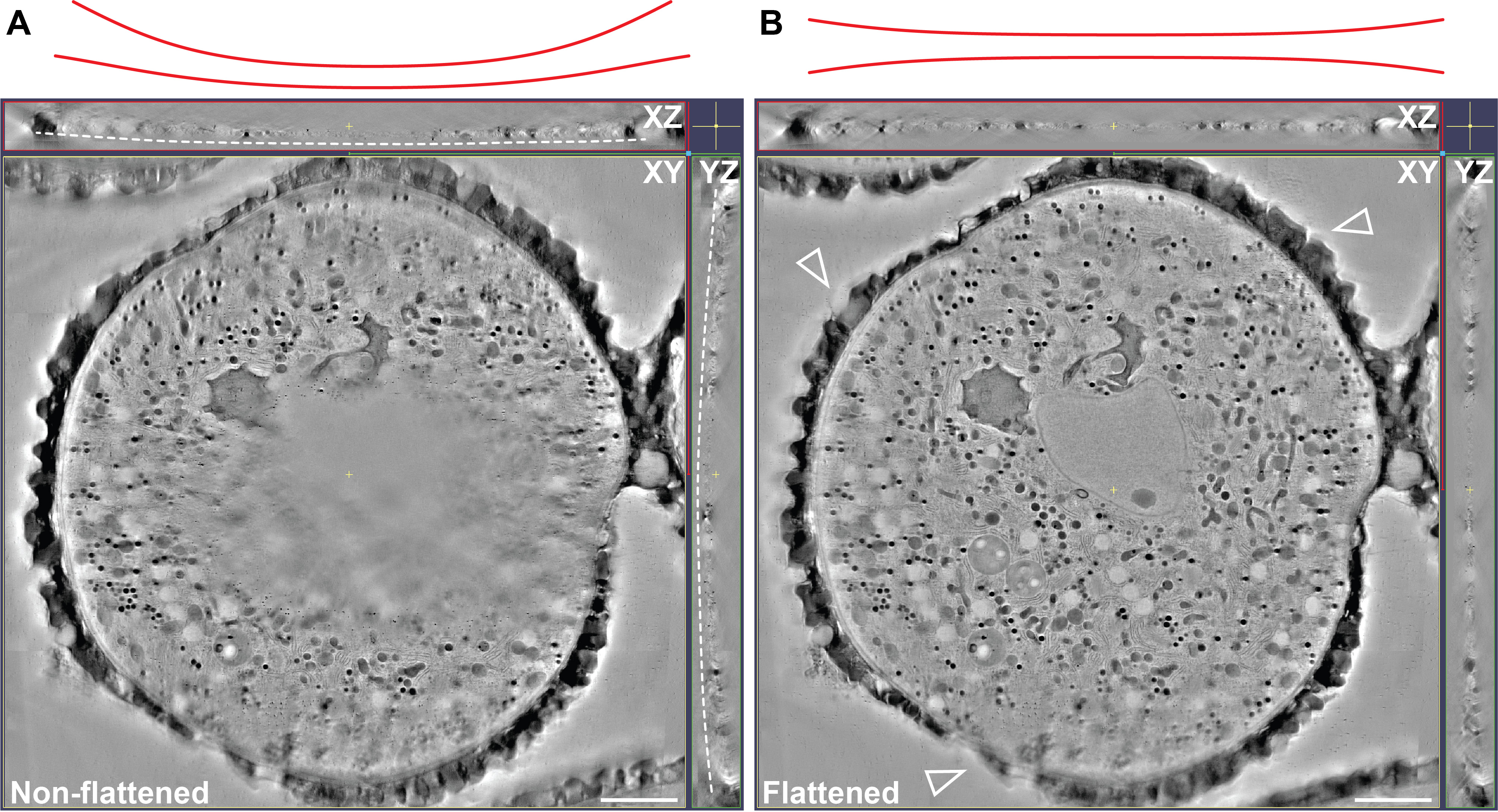


**Figure S1. Tomographic slices and the impact of section warping on reconstruction quality**

**(A)** Tomographic slice view of a reconstructed room-temperature (RT) electron tomogram without Flatten processing. White dash lines indicate the warping trend of the plastic section. The side (XZ) view of the reconstructed volume is illustrated in red lines (as surface boundaries of the section) at the top.

**(B)** Tomographic slice view of the same RT electron tomogram after Flatten processing. Arrowheads indicate severe blurry and phantom of the pollen grain margin. The side (XZ) view of the reconstructed and processed volume is illustrated in red lines (as surface boundaries of the section) at the top. Note the remaining thickened and blurred lateral regions of the section.

Scale bars, 2 μm.


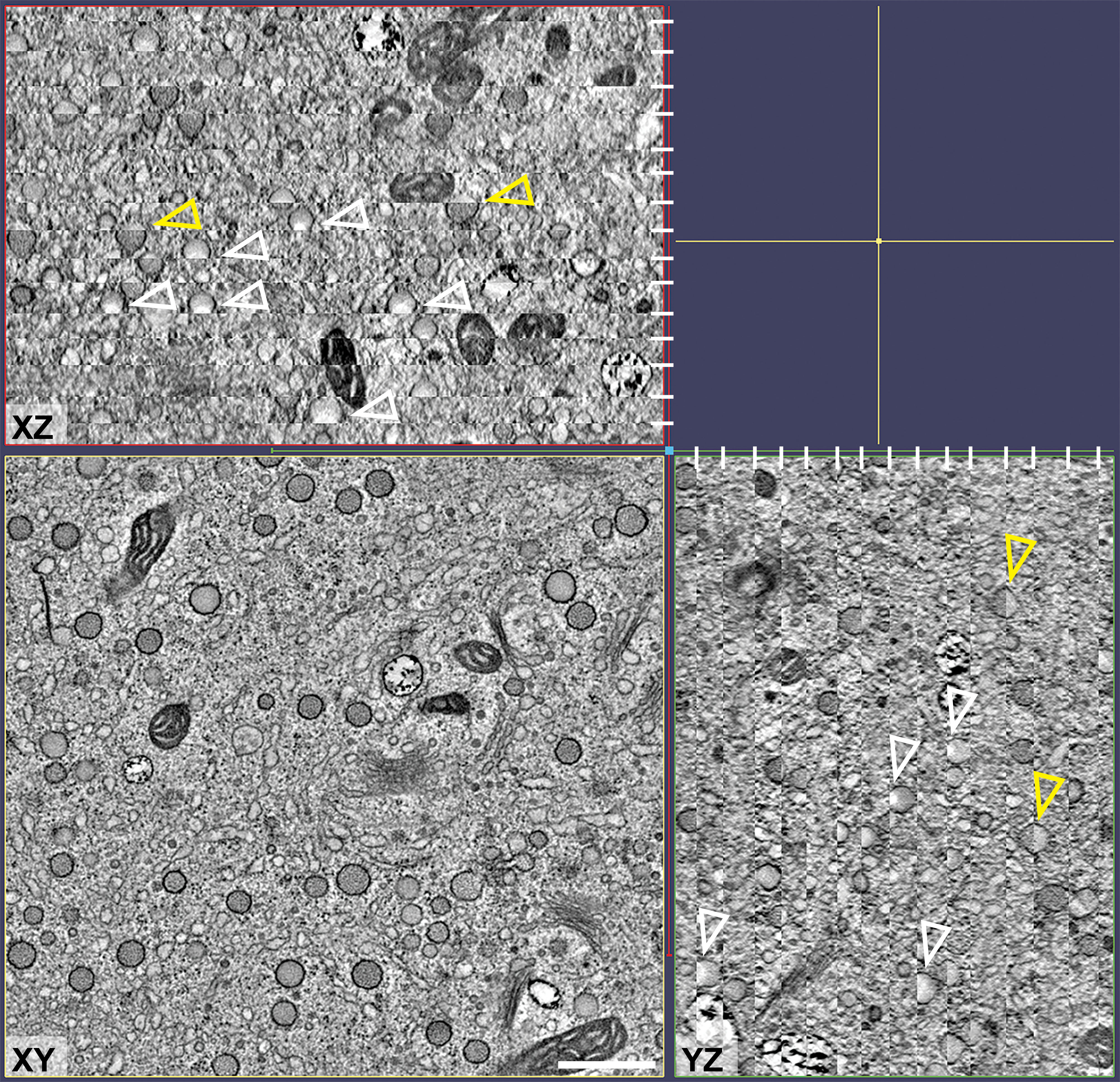


**Figure S2. Tomographic slices of a joined tomogram covering the tobacco pollen tube tip region**

Ultrathin sections of a *Nicotiana tabacum* pollen tube were imaged by room-temperature electron tomography (RT-ET) at a 4.32-nm pixel size. Side views (XZ and YZ) of the joined volume show 16 serial sections. Dashes indicate the interfaces of adjacent sections. Note the bright cone-shaped ‘hollow’ regions near the section surfaces indicated by white arrowheads. These hollows consistently appear on the same surface across sections. The yellow arrowheads indicate the distinct lumen densities between the two halves of the same vesicle embedded in two adjacent sections. Scale bar, 500 nm.


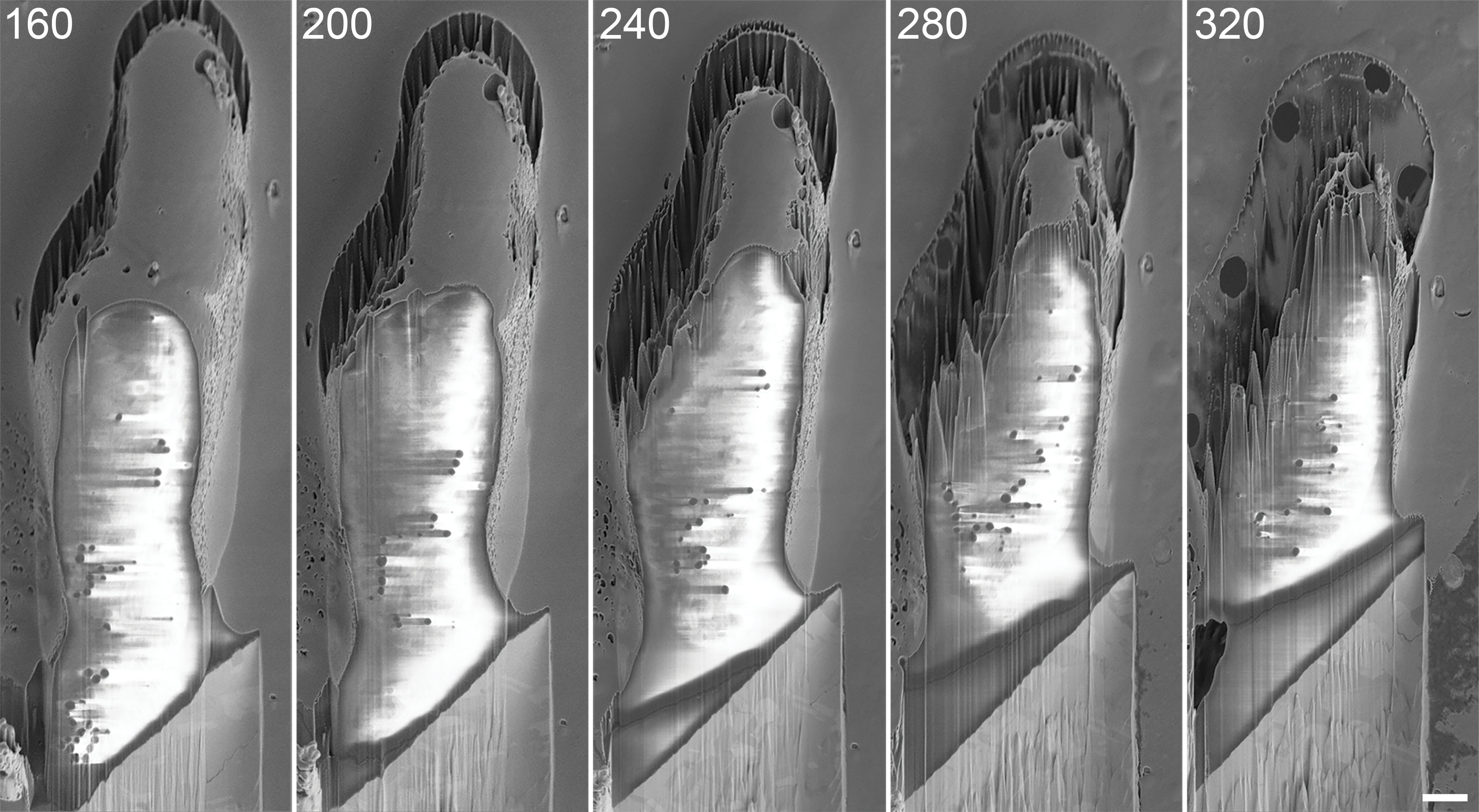


**Figure S3. Worn protective coating at the interface of the cell and the grid film**

Sequential images by scanning electron microscopy (SEM) imaging of the focused ion beam (FIB)-milled *Arabidopsis* pollen tube show the interface between the cell and the grid film is prone to damage during the FIB-SEM imaging. Sequential numbers of the slices are labeled at each upper left corner.

Scale bar, 2 μm.


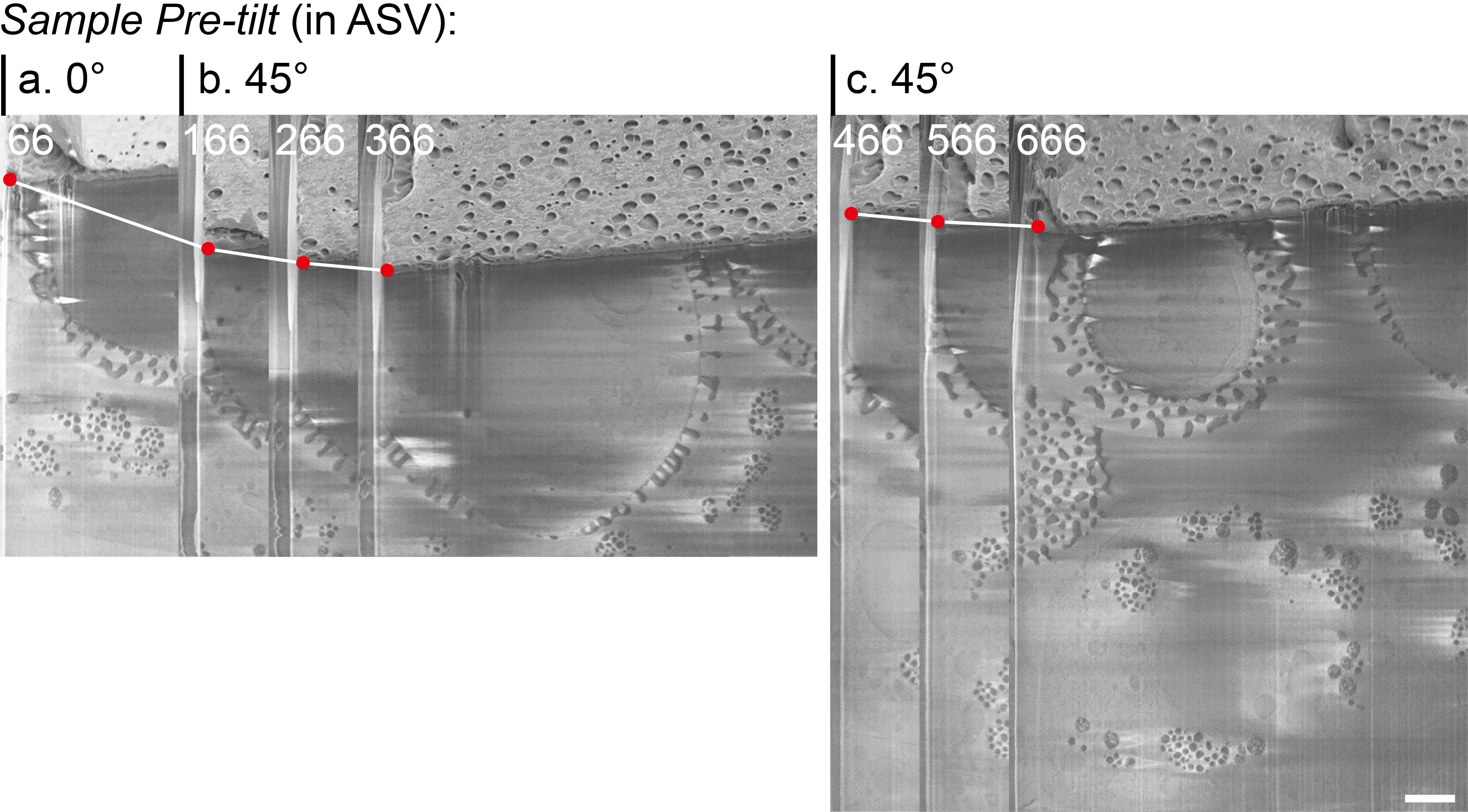


**Figure S4. Compensation for the image Y-shift by *Sample Pre-tilt* input**

Sequential images by scanning electron microscopy (SEM) imaging of the focused ion beam (FIB)-milled *Arabidopsis* pollen grain show the progressive Y-axis shift as the number of milling-imaging loops increases. This shift can be alleviated by resetting proper *Sample Pre-tilt* in Auto Slice & View (ASV) software. Sequential numbers of the slices are labeled at each upper left corner. Ion beam is normal to the sample/carrier top surface.

Before slice 166, *Sample Pre-tilt* was set 0° in the *MILLING* tab in ASV. After slice 166, *Sample Pre-tilt* was set 45° in ASV. After slice 466, selected scan area was rescaled to cover larger area downwards to ensure the interested pollen grain stay in the scan area without supervision.

Scale bar, 2 μm.

**
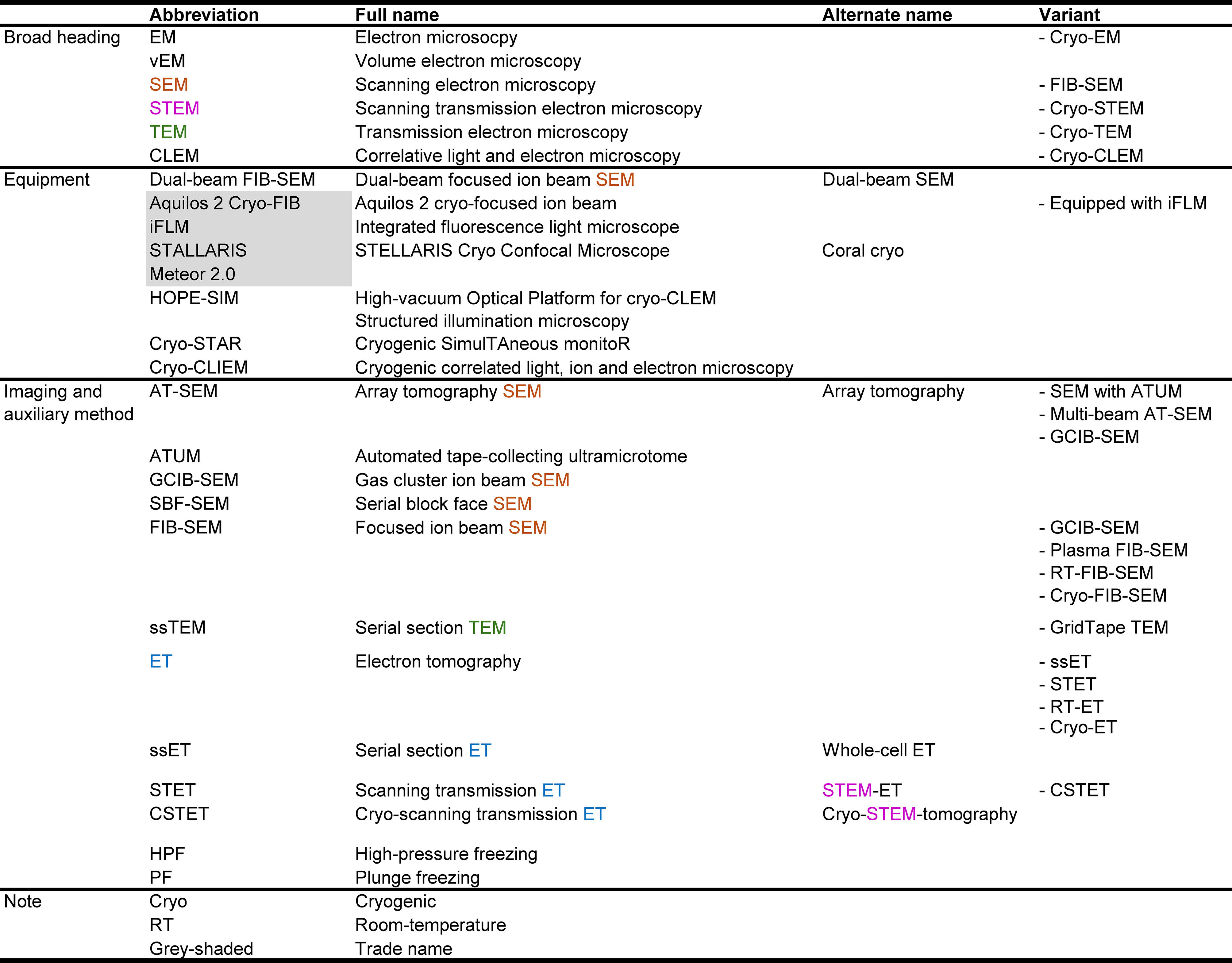
Table S1. Glossary of terms**

**Movie S1. Aligned serial scanning electron micrographs of the slices of the *Arabidopsis* pollen grain (Corresponding to Figure 3G)**

The frames show the pollen grain being targeted in ROI1 in Figure 3G. Total frame/slice number in the movie is 672. Total frame/slice number in the ASV program was 3398. Scale bar 5 μm.

**Movie S2. Aligned serial scanning electron micrographs of the slices of the germinating tobacco pollen tube (Corresponding to Figure 4H)**

Total frame/slice number in the movie is 695. Total frame/slice number in the ASV program was 2773. Scale bar 2 μm.

**Movie S3. Electron tomogram of the lamella prepared from a plunge-frozen *in vitro*-cultured *Arabidopsis* pollen tube (Corresponding to Figure 5G)**

Scale bar 200 nm.

**Movie S4. Electron tomogram of the lamella prepared from high-pressure-frozen *Arabidopsis* anther (Corresponding to Figure 6L)**

Scale bar 200 nm.

**Movie S5. Aligned serial cryo-scanning electron micrographs of the slices of the *Arabidopsis* pollen tube (Corresponding to Figure 7G)**

Total frame/slice number in this movie is 225. Total frame/slice number in the ASV program was 470. Scale bar 2 μm.

**Movie S6. Aligned serial cryo-scanning electron micrographs of the slices of the *Arabidopsis* pollen grain (Corresponding to Figure 8H)**

Total frame/slice number in this movie is 270. Total frame/slice number in the ASV program was 958. Scale bar 2 μm.
